# Genomic and Metabolic Characteristics of the Pathogenicity in *Pseudomonas aeruginosa*

**DOI:** 10.3390/ijms222312892

**Published:** 2021-11-29

**Authors:** Telma de Sousa, Michel Hébraud, Maria L. N. Enes Dapkevicius, Luís Maltez, José Eduardo Pereira, Rosa Capita, Carlos Alonso-Calleja, Gilberto Igrejas, Patricia Poeta

**Affiliations:** 1 Department of Genetics and Biotechnology, University of Trás-os-Montes and Alto Douro (UTAD), 5000-801 Vila Real, Portugal; telmas@utad.pt (T.d.S.); gigrejas@utad.pt (G.I.); 2 Microbiology and Antibiotic Resistance Team (MicroART), Department of Veterinary Sciences, University of Trás-os-Montes and Alto Douro (UTAD), 5000-801 Vila Real, Portugal; 3 Functional Genomics and Proteomics Unit, University of Trás-os-Montes and Alto Douro (UTAD), 5000-801 Vila Real, Portugal; 4 Associate Laboratory for Green Chemistry (LAQV), Chemistry Department, Faculty of Science and Technology, University Nova of Lisbon, 2829-516 Lisbon, Portugal; 5 Institut National de Recherche pour l’Agriculture, l’Alimentation et l’Environnement (INRAE), Metabolomic and Proteomic Exploration Facility (PFEM), 63122 Saint-Genès-Champanelle, France; michel.hebraud@inrae.fr; 6 Université Clermont Auvergne, INRAE, UMR Microbiologie Environnement Digestif Santé (MEDiS), 60122 Saint-Genès-Champanelle, France; 7 Faculty of Agricultural and Environmental Sciences, University of the Azores, 9700-042 Angra do Heroísmo, Portugal; maria.ln.dapkevicius@uac.pt; 8 Institute of Agricultural and Environmental Research and Technology (IITAA), University of the Azores, 9700-042 Angra do Heroísmo, Portugal; 9 Department of Veterinary Sciences, University of Trás-os-Montes and Alto Douro (UTAD), 5000-801 Vila Real, Portugal; lmaltez@utad.pt (L.M.); jeduardo@utad.pt (J.E.P.); 10 Veterinary and Animal Research Centre, University of Trás-os-Montes and Alto Douro (UTAD), 5000-801 Vila Real, Portugal; 11 Department of Food Hygiene and Technology, Veterinary Faculty, University of León, E-24071 León, Spain; rosa.capita@unileon.es (R.C.); carlos.alonso.calleja@unileon.es (C.A.-C.); 12 Institute of Food Science and Technology, University of León, E-24071 León, Spain

**Keywords:** *Pseudomonas aeruginosa*, pathogenicity, multidrug-resistant, biofilm, virulence factors

## Abstract

In recent years, the effectiveness of antimicrobials in the treatment of *Pseudomonas aeruginosa* infections has gradually decreased. This pathogen can be observed in several clinical cases, such as pneumonia, urinary tract infections, sepsis, in immunocompromised hosts, such as neutropenic cancer, burns, and AIDS patients. Furthermore, *Pseudomonas aeruginosa* causes diseases in both livestock and pets. The highly flexible and versatile genome of *P. aeruginosa* allows it to have a high rate of pathogenicity. The numerous secreted virulence factors, resulting from its numerous secretion systems, the multi-resistance to different classes of antibiotics, and the ability to produce biofilms are pathogenicity factors that cause numerous problems in the fight against *P. aeruginosa* infections and that must be better understood for an effective treatment. Infections by *P. aeruginosa* represent, therefore, a major health problem and, as resistance genes can be disseminated between the microbiotas associated with humans, animals, and the environment, this issue needs be addressed on the basis of an One Health approach. This review intends to bring together and describe in detail the molecular and metabolic pathways in *P. aeruginosa*’s pathogenesis, to contribute for the development of a more targeted therapy against this pathogen.

## 1. Introduction

One of the current greatest global public health problems is the pathogenesis associated with the emergence of generalized infectious bacteria [1]. *Pseudomonas aeruginosa* has the ability to infect not only humans, but also animals and plants. Given its role as an important human pathogen, several virulence factors have been studied, as well as their regulatory systems [2]. This pathogen is armed with a wide set of virulence factors, allowing it to harm host cells and modulate human adaptive immune mechanisms, thus causing the appearance of new infections [3]. The T3SS system is one of its main virulence factors, and a very complex matrix of environmental signals is integrated to tightly control the injection of proteins into host cells, where they are directed to specific compartments and manipulate a number of cell processes [4]. However, the pathogenesis of this bacterium is not exclusively focused on the emergence of new infectious diseases, but also on biofilm formation and on the appearance of new antibiotic-resistant strains, or on the increase in prevalence of already known strains [5]. The acquisition of resistance to two or more classes of antibiotics by pathogenic bacteria is denominated as multidrug-resistance (MDR) [6]. *P. aeruginosa* is one of the bacterial species that have developed an alarming number of multidrug-resistant strains. These strains are associated with significant morbidity and mortality and they are responsible for 10% of nosocomial infections [7]. This review aims to provide an overview of the metabolic pathways underlying the pathogenicity of *P. aeruginosa* and to describe minutely mechanisms such as its different virulence factors and their multiple regulatory systems, as well as the biofilm formation and resistance to different antibiotics. The purpose of this review is also to provide a better understanding of these mechanisms and to support further research into alternatives to combat this pathogen.

## 2. *Pseudomonas aeruginosa*

*Pseudomonas aeruginosa* is a ubiquitous bacterium, present both in terrestrial and aquatic ecosystems. As a result, it can be found in many foodstuffs but also in hospital environments. *P. aeruginosa* is considered to be a bacterium of major medical importance due to its great ability to adapt to different environments, but also because of its ability to cause chronic infection in vulnerable people [8]. The first report describing the genus *Pseudomonas* came from the German botanist Walther Migula in the late 19th century. He visualized mobile cells with spores, such as the nanoflagellate *Monas* spp. Thus, the name *Pseudomonas* was generated from the identification of this false or “pseudo” *Monas* spp. nanoflagellate [9]. However, the specific name “*aeruginosa*” was described earlier in 1872 by Schroeter because of the color of the colonies on certain media, resembling copper rust or verdigris color, hence being green. Schroeter attached the name to the genus *Bacterium*, to give *Bacterium aeruginosum*, and later, Migula transferred the species to the newly described genus *Pseudomonas* [10].

*P. aeruginosa* is a gram-negative, bacillus-shaped bacterium (size from 0.5 to 3.0 µm), with an aerobic metabolism and a single flagellum that helps in move. It is a non-fermentative bacterium which, in aerobic situations, uses the glycolytic pathway for glucose degradation, with oxygen as its final electron acceptor. However, under anaerobic conditions, nitrogen can be used as an electron acceptor. Furthermore, ATP acquisition is achieved by the action of several membrane ATPases reusing ADP and H^+^ from previous reactions. It should be noted that sometimes, aromatic compounds like phenolsulfates and phenylalanine can be considered as putative substrates for this species’ catabolic activity [11]. This species can survive in a wide range of environmental conditions (mostly regarding their chemical composition), it can use a variety of different carbon, nitrogen and energy sources, but it has a rather limited range of growth temperatures [10].

### 2.1. Bacterial Pathogenesis

*P. aeruginosa* is adaptable to different environments and metabolically versatile, covering a wide variety of habitats, including the human body, soil, water, hospital environment, drains, but also other water-rich locations (swimming pools), as well as non-household environments (such as river water), and can also be isolated from vegetables [12,13,14,15]. This is due to the minimum nutritional requirements necessary for the growth of this bacterium and its ability to survive under a variety of environmental and physical conditions [11,14]. The dissemination of new infections that come from the environment and hospitals requires special attention in order to minimize the chance of nosocomial infection by this pathogen [13,16].

The ubiquity of *P. aeruginosa* in nature comes from its natural ability to use several mechanisms to resist against other organisms. For example, when using the type III secretion system to directly inject cytotoxic effector proteins into the cytosol of eukaryotic cells, a polysaccharide-encased community (a biofilm) of *P. aeruginosa* can be formed and this community can resist to predation by protozoa [17,18].

The infection potential of *P. aeruginosa* comes in part from the presence of virulence factors and the ability to metabolize many antibiotics (encoded in genes that are collectively organized in genomic islands), consequently, promoting infectivity potential [19,20,21]. In general, virulence factors can be classified as cell- associated or extracellular virulence factors [22,23], both being secreted via secretion systems. Cell-associated virulence factors are important for the bacterial colonization process and they are secreted and bound to the cell envelope (inner or outer membrane) [18]. On the other hand, extracellularly virulence factors are released by *P. aeruginosa* after colonization and they are secreted into the extracellular medium (exoproteins) [18].

### 2.2. Clinical Impact of Pseudomonas aeruginosa

*P. aeruginosa* is an opportunistic pathogen. It lives as a commensal and environmental organism, but can occasionally change to a pathogenic state, causing infections that are difficult to treat [24]. For example, *P. aeruginosa* is the major cause of infections such as ventilator-associated pneumonia, urinary tract, bloodstream, and chronic infections [25,26,27]. In immunocompromised hosts, like AIDS or diabetes mellitus patients, bacteremia caused by this bacterium is a common complication [26]. In animals, *P. aeruginosa* causes diseases in both livestock and pets. For example, it causes otitis and urinary tract infections in dogs, mastitis in dairy cows, hemorrhagic pneumoniae in fur-bearing animals such as mink or foxes, and endometritis in horses [28].

This bacterium displays a natural resistance to many classes of antibiotics and its capacity to rapidly develop new ones during treatment represents one of the most common reasons for therapeutic failures [29]. *P. aeruginosa* is one of the six “ESKAPE” pathogens belonging to the World Health Organization “priority pathogens” list of antibiotic resistance [1]. The resistance rate of *P. aeruginosa* to antibiotics is increasing worldwide and that is why it is necessary to increase monitorization of infections in animals and humans in order to minimize possible transfers between hosts [28].

*P. aeruginosa* can also develop and grow in a sessile community structure that provides protection against antibiotics, host defense mechanisms, desiccation, ultraviolet light, and disinfectants [30]. Given its resistance to antibiotics, the opportunistic, and resilient character, it has become one of the most concerning issues of pathogenesis nowadays and has drawn the attention of many researchers in the quest to obtain more knowledge and understanding [7,31,32].

## 3. Genome Structure in *Pseudomonas aeruginosa*

**Figure 1:**
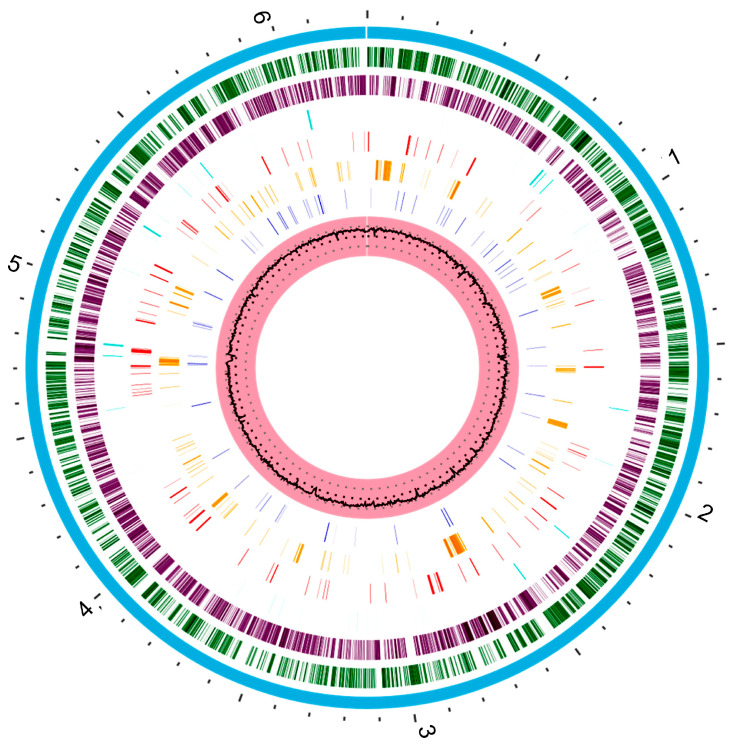
The circular representation of the *P. aeruginosa* PAO1 genome generated with www.patricbrc.org (accessed in 8 May 2021). This genome contains about 6.3 Mbp. The pink circle that contains a black circle in the middle is the G + C content percentage (66.6%). The dark blue bars represent a drug target. Orange bars represent genes encoding virulence factors and the red bars genes encoding antimicrobial resistance. Cyan bars represent non-coding regions. The purple and dark green circle represent the reverse and forward coding regions of the genome, respectively. Finally, the blue circle that covers all regions represents the entire chromosome.

The genetic repertoire of *P. aeruginosa* reflects the adaptative character of this bacterial species [11]. The metabolic versatility is provided by genes encoding enzymes that participate in the metabolic pathways, transcriptional regulators, and regulatory systems [33]. The whole genome sequence of *P. aeruginosa* strain type PAO1 was published by Stover and other collaborators in 2000. Thereafter, several strains have had their whole genomes sequenced [34]. The *P. aeruginosa* PAO1 genome is used as a reference for comparing with the genomes of other strains [34]. *P. aeruginosa* PAO1 has a very large and complex genome, around 6.3 Mbp (G + C content 66.6%), encoding 5700 genes, including 5584 predicted open read frames (ORFs) [34,35]. In addition, it is estimated that 150 of the genes identified in *P. aeruginosa* PAO1 encode outer membrane proteins related to adhesion, movement, antibiotics, and virulence factor output (Figure 1) which represents a much higher number when compared to other genomes [36].

*P. aeruginosa* has a mosaic genome, composed of many core genes interspersed by strain-specific blocks of genes [37,38]. As a result of comparative genomic studies within the species, the genome of *P. aeruginosa* has been classified in three groups depending on its characteristics: the core genome, the accessory genome, and the pan-genome [18,35,37,38,39].

The core genome defines the region of the genome possessed by almost all *P. aeruginosa* strains, interspersed with “accessory” genomic elements that are present in some, but absent in other strains of the same species [33,35]. The core genome regions represent approximately 90% of the total *P. aeruginosa* genome, display low levels of genetic diversity (0.5–0.7%), contain the majority of genes with housekeeping functions, and do not tolerate excessive changes over short evolutionary periods [40,41,42].

The accessory genome of *P. aeruginosa* consists of non-conserved, variable-length stretches of DNA, generally located in extrachromosomal elements, and blocks of inserted DNA in certain loci [33,43]. These DNA segments tend to cluster at certain loci, rather than being randomly distributed throughout the core genome [40]. These loci are often referred as genomic islands (>10 kb) [43]. This part of genome is very relevant for its clinical implications, since it harbors genes that encode proteins with homologies to virulence factors and genes that encode resistance to various classes of antibiotics [39,44]. For example, genomic island of *P. aeruginosa* PAPI-2 contain the gene encoding ExoU, a type-III secreted effector protein linked to increased virulence in animal models and human patients [35]. The intrinsic determinants of antibiotic resistance in *P. aeruginosa*, such as efflux pumps and a β-lactamases, are located in the core genome. However, genes of acquired antibiotic resistance are present in the accessory genome [18,31]. Indeed, the dissemination of multidrug-resistant strains is often due to the resistance genes present in the accessory genome [39]. Therefore, identification of these accessory genomic elements in *P. aeruginosa* has a major impact on the study of the evolution, adaptation, and infectious potential of this organism [35].

The general architecture of the *P. aeruginosa* pan-genome can be represented as a circular chromosome with polymorphic strain-specific segments, flanked by conserved genes referred to as anchors [40]. Basically, this genome consists of genes within the core genome and accessory genome, with the latter containing all “dispensable”, strain-specific genes present only in a subset of strains [45]. The size of the pan-genome currently exceeds 10 Mbp, whereas the core genome is roughly 5.84 Mbp, representing 89.7% of the total genome, and the accessory genome is about 727 kbp, representing, in average, 11.1% of the total genome [35]. The *P. aeruginosa* pan-genome contains approximately 54,272 genes, 665 of which are core genes, 26,420 are accessory genes, and 27,187 are unique genes (present in one strain only) [39].

## 4. Metabolic Pathways Associated with Pathogenicity

With new and robust results of genome sequencing, an increasing interest in the *P. aeruginosa* genome has taken place, mainly with the purpose of better understanding its adaptive mechanisms and multi-omics interactions [46]. Studies have shown that, although the *P. aeruginosa* genome is well conserved and shows moderate genetic variability, non-synonymous mutations, and recombination events play an important role in phenotypic diversification [47]. Genes that encode for aerobic respiration, denitrification, and anaerobic fermentation, found in the core genome, have been identified in all strains of *P. aeruginosa*, highlighting the metabolic versatility of this bacterium [48]. Proteins involved in regulation, transport, virulence and resistance also contribute to the high nutritional versatility and adaptive capacity of this bacterium [26]. Thus, the study of extracellular proteins is of high interest due to their essential roles in bacterial lifestyle [49]. Currently, although antibiotic pressure, airway heterogeneity and the natural versatility of *P. aeruginosa* play an important role in its diversification, the exact mechanisms that drive adaptability and diversification in this species are still not fully understood [2,50,51].

### 4.1. Virulence Factors

#### 4.1.1. Secretion Systems

**Figure 2:**
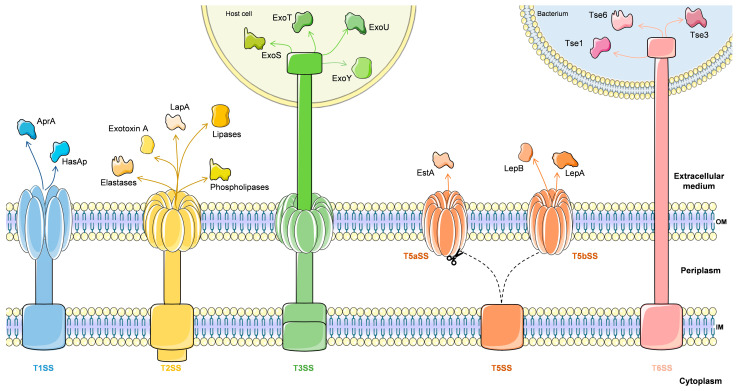
The secretion systems in *P. aeruginosa***.** Five secretion pathways are present in *P. aeruginosa*: The T3SS and T6SS transport proteins from the bacterial cytoplasm to the cytosol of the target cell. On the other hand, the T1SS, T2SS and T5SS transport proteins from the bacterial cytoplasm to the extracellular medium.

*P. aeruginosa* possesses a series of complex secretion systems, which deliver virulence factors such as toxins, elastases, lipases, and proteases, either to the extracellular environment or within the cytosol of the host cell [52]. *P. aeruginosa* contains five secretion systems: type I, type II, type III, type V and type VI (Figure 2) [53].

Type I secretion system (T1SS) is one of the simplest bacterial secretion systems described to date. T1SS require three components: an outer membrane (OM) protein, an ABC (ATP-binding cassette) transporter, which is inserted in the inner membrane (IM) and provides energy to the transport process, and an adaptor protein connecting these two components in the periplasm [54]. In *P. aeruginosa*, two types of T1SS exist. One of these types is the HasAp system, that consists of an ABC transporter (HasD), adaptor (HasE), and OMF (HasF) [52,54]. The HasAp protein is a hemophore, with the ability to bind heme from hemoglobin. This protein is considered crucial component for the survival of *P. aeruginosa* and is especially important during the early stages of infection, where iron is scarce, as it can allow the acquisition of heme iron from hemoglobin [54]. The other type of T1SS is the Apr system, which involves an ABC transporter (AprD), adaptor (AprE), and OMF (AprF), and is implicated in the extracellular secretion of the alkaline protease AprA and of AprX [54,55], which is a virulence factor involved in various infections caused by this bacterium [56].

Type II secretion system (T2SS) is a very versatile system. Being widely preserved in Gram-negative bacteria, its sole purpose is to promote the transport of large multimeric proteins, previously folded in the periplasm, by the outer membrane [57]. T2SS uses a two-stage process for the delivery of extracellular proteins: the first one involves the Sec- or Tat-dependent delivery from the cytoplasm into the periplasm, and the second stage involves T2SS complex-mediated further secretion into the extracellular space [58]. In *P. aeruginosa*, there are two different T2SS types: the Xcp (extracellular protein) system can secrete at least 14 proteins with different functions, such as proteases and lipases, and the Hxc (homologous to Xcp) system, only functional in phosphate-limiting growth conditions [59].The latter system can only secrete one protein, LapA, an alkaline phosphatase. The Xcp system is encoded by 11 genes organized into two divergent operons, xcpP to Q and xcpR to Z. Moreover, there is a 12th gene, xcpA/pilD, located beyond the 2 xcp operons [53,60]. Some examples of secreted proteins include elastase (LasB, LasA), phospholipases, and lipases (PlcN, PlcH, LipA and LipC), as well as exotoxin A (ToxA), all of which playing major roles during infection. Elastase leads to the degradation of the connective tissue in the lungs, which is mainly composed of elastin [61]. Also affecting the pulmonary system, phospholipases and lipases degrade surfactants and modify immune function in these organs [62,63]. On the other hand, exotoxin A is one of the most important virulence factors in *P. aeruginosa,* because it inhibits protein synthesis by ADP-ribosylation of cell elongation factor-2 in the host cell, consequently leading to cell death [64]. A study carried out by Mapipa and his collaborators demonstrated that samples of *P. aeruginosa* in hospital wastewater harbored virulence traits through the identification of genes that conferred this virulence on them, such as *popB*, *lasB*, *lasA and toxA.* This demonstrates a serious public health problem since these virulence factors can stimulate the adaptability of these bacteria to the environment and exert pathogenicity on the susceptible host [65].

The type III secretion system (T3SS) forms a needle complex which allows the injection of effector proteins from the bacterial cytosol to the extracellular environment [4], and is also related to the flagellar assembly machine [53]. This involves a high degree of complexity; therefore, this system has thirty-six genes, encoded in five operons, that are organized and clustered together in the *P. aeruginosa* chromosome. These genes are implicated in the biogenesis and regulation of the type III secretory-translocation machinery [53]. One of the important components of T3SS in *P. aeruginosa* is the production of at least four effector proteins: ExoS, ExoT, ExoU, and ExoY, that cause cytotoxicity at different levels [66]. The ExoT and ExoS are homologous bifunctional effectors and are both ADP-ribosyltransferases (ADPRT) and GTPase-activating protein (GAP) activity. These effector proteins seem to target host signaling pathways, specifically through ADP-ribosylation of Ras, affecting the function of the host cell [67,68,69]. ExoU is an effector with lipase activity that has broad substrate specificity, causing tissue destruction. ExoY is an adenylate cyclase that increases cAMP levels intracellularly and induces rounding of cells by disrupting the actin in their cytoskeleton [70].

In *P. aeruginosa*, type III secretion is regulated at two levels: transcription of T3SS genes and initiation of secretion. Both are switched on upon contact with host cells, leading to the production of a large amount of secreted type III components [71,72]. Transcription of T3SS genes is induced under limiting calcium conditions or shortly after contact of these bacteria with their host cells [53]. Transcription of T3SS genes is controlled by the ExsA master regulator, which is a member of the AraC family of transcriptional activators [73]. ExsA binds to a nucleotide sequence upstream of the RNA polymerase binding site of T3SS promoters [74]. ExsA autoregulates its expression by binding to the promoter region of *exsCEBA* (coding region of the T3SS regulatory complex) [75]. Downstream of this regulatory complex operon is the anti-activator *exs*D gene which inhibits exsA-dependent transcription [72]. The exsC, an anti-anti-activator, binds directly to exsD in under low calcium conditions, which promotes the inactivation of ExsA, thus allowing interaction with the promoter region [76]. There is also a secreted anti-anti-anti-activator, ExsE, that binds to ExsC preventing it from inhibiting ExsD [77]. In general, when ExsE is secreted, it remains intracellularly, which favors the formation of ExsE-ExsC and ExsA-ExsD complexes, thus avoiding ExsA-dependent transcription. On the other hand, in the absence of ExsE, no binding to ExsC takes place. Thus, ExsC sequesters ExsD, allowing ExsA to activate the transcription of T3SS genes [76,77]. Besides this pathway, the T3SS regulon is under control by several upstream regulators, including cAMP biosynthesis [42], a global regulator, Vfr, and ResT/LadS/GacAS two-component regulatory systems [74,78].

Another trigger for the expression of genes in the T3SS system is a spermidine transporter, SpuDEFGH, which allows *P. aeruginosa* to have the ability to detect and sense the signaling molecules in the fluid of its host’s tissues and, consequently, induces the expression of T3SS genes [79]. When the spermidine transporter genes are deleted, host-cell-dependent induction of T3SS genes is inhibited, and so are bacterial cytotoxicity and expression of *exsCEBA* and effector genes induced by calcium depletion [74]. Spermidine is an organic polycation, belonging to the group known as polyamines, which play important roles in stress response, acid tolerance, and antimicrobial resistance [80,81,82]. Some studies show that polyamines can either induce resistance to various classes of antibiotics or increase susceptibility. For example, in *P. aeruginosa*, polyamines can induce resistance to aminoglycosides and quinolone antibiotics. On the other hand, it was also shown that exogenous addition of spermidine and other polyamines increases the susceptibility to β-lactam antibiotics, trimethoprim, chloramphenicol, and nalidixic acid, by inducing the expression of the efflux pump *oprH-phoPQ* operon and of an LPS modification operon [81,83]. Thus, the identification of the spermidine signal and its transporter could provide a good approach to control infections by *P. aeruginosa*.

The type V secretion system (T5SS) is the simplest secretion pathway described so far. The T5SS consists of autotransporters (AT or T5aSS) and two-partner secretion systems (TPS or T5bSS) [53]. Basically, in this system, proteins are transported to, and remain associated with, the outer face of the outer membrane, or are released into the extracellular medium after proteolytic cleavage [84]. The T5aSS proteins are modular proteins consisting of a signal peptide at the extreme N terminus and a β-domain of 12 β-strands at the C terminus, connected by a “passenger” domain that harbors the catalytic domain of the protein. The signal peptide crosses the inner membrane and is subsequently cleaved. In the periplasm, the C terminus domain is inserted into the outer membrane and forms a β-barrel. This process results in the exposure of the passenger domain on the surface of the bacteria or its release into the extracellular medium after cleavage either by autoproteolysis or by a protease [53]. In *P. aeruginosa*, the EstA is the only characterized T5aSS [85]. EstA has an autotransporter esterase activity and is located on the outer side of the outer membrane. It has been shown to be important for the production of rhamnolipid, which in turn affects cell motility and biofilm formation [86]. The T5bSS is very similar to T5aSS, except that the passenger domain is called TpsA and the β domain TpsB, and both are independent proteins [87]. In general, the TpsA and TpsB proteins cross the inner membrane through the Sec export pathway. TpsA is converted to a pro-protein after maturation of its signal peptide. In the outer membrane, TpsB recruits TpsA through POTRA domains that interact with the TpsA T5bSS motif. The pro-TpsA protein is subsequently cleaved to form a mature TpsA protein, which may remain anchored to the surface of the bacteria or be released into the extracellular milieu [53]. In the genome of PAO1, six groups of T5bSS were noted, among which LepA/LepB, that secretes a protease influencing the host’s response to bacterial infection. CdrA/CdrB is responsible for transporting CdrA to the outer membrane, promoting biofilm formation and auto-aggregation in liquid culture [88,89].

The type VI secretion system (T6SS) is also a needle-like complex and is widely spread in Gram-negative bacteria [90]. In this secretion system, proteins without a signal peptide, denominated by Hcp (hemolysin-coregulated protein) and VgrG (valine-glycine repeat), require a functional T6SS for their release into the extracellular medium [91]. The crystal structure of the *P. aeruginosa* Hcp1 protein reveals a hexameric ring structure, homologous to the tube domain of the T4 phage tail (gp19 protein). On the other hand, the conserved N-terminal region of VgrGs shares similarities with the T4 phage proteins (gp5 and gp27 proteins). The gp5 and gp27 multimers constitute the bacteriophage tail spike used for puncturing the bacterial envelope and to inject DNA into the cytoplasm [53]. Thus, it was proposed that recognition of the target cell and effective delivery occur in a similar way to bacteriophage entry in the host cell [92]. Other components of T6SS are: a ClpV ATPase, a FHA regulatory domain protein that is the target of a pair of Ser/Thr kinase and phosphatase, DotU- and IcmF-like proteins, and an outer membrane lipoprotein [53].

In *P. aeruginosa*, there are three loci encoding T6SS components (HSI-I, HSI-II and HSI-III), which constitute a set of 15–20 genes involved in interaction with other bacteria and the environment, similar to the T3SS [93]. At least six effector proteins (Tse1-6) are secreted through the T6SS. Tse1 and Tse3 are inserted into the periplasm and hydrolyse peptidoglycan, consequently causing the lysis of bacterial cells. Tse2 arrests the growth of both prokaryotic and eukaryotic cells. Tse4, Tse5 and Tse6 function as antibacterial effectors [94,95]. The HSI-I needle is a dynamic contractile structure, much like a phage tail, projecting from the cytosol onto the bacterial surface; it is regulated by two-component systems and sRNAs [95,96]. HSI-II increases bacterial internalization in epithelial cells, while HSI-III plays important roles in pathogenesis [97]. Regulators and expression of the HSI-II or HSI-III are affected by transcriptional factor MvfR, a quorum sensing (QS) regulator [90]. In addition, in *P. aeruginosa*, HSI-II and HSI-III play important roles in the infection process in animals and plants [97].

This diversity of secretion systems may have resulted from an evolutionary process that optimized various biological functions in *P. aeruginosa.* Although this pathogen lacks some secretion systems, such as T4SS, it still has a great diversity of systems. All of these systems enable *P. aeruginosa* to cause various types of infections in various human and animal tissues, since they endow it with numerous virulence factors.

#### 4.1.2. Motility

**Figure 3:**
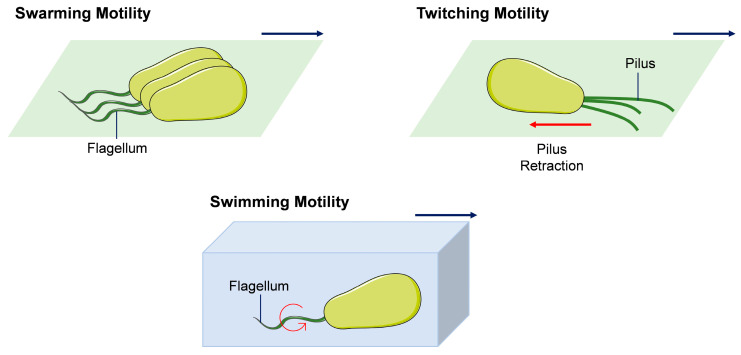
The three types of bacterial motility which *Pseudomonas aeruginosa.* The cell’s direction of movement is represented by dark blue arrows, and the motors that drive the movement are represented by red arrows.

In bacteria, motility plays a fundamental role in the colonization of abiotic surfaces (e.g., medical devices) and biotic surfaces (e.g., damaged tissues), as well as in the spreading of bacteria across the surface [98], thereby contributing to virulence. Motility contributes to the formation of biofilm and, consequently, the formation of problematic bacterial communities, with increased resistance to antimicrobial agents [99]. In *P. aeruginosa*, cell surface organelles, flagella, and pili are used for motility. The surface-associated, twitching motility, is dependent on the extension and retraction of type IV pili. The flagellum allows this bacterium to swim in liquid media and swarm across a solid surface (Figure 3) [100,101].

**Figure 4:**
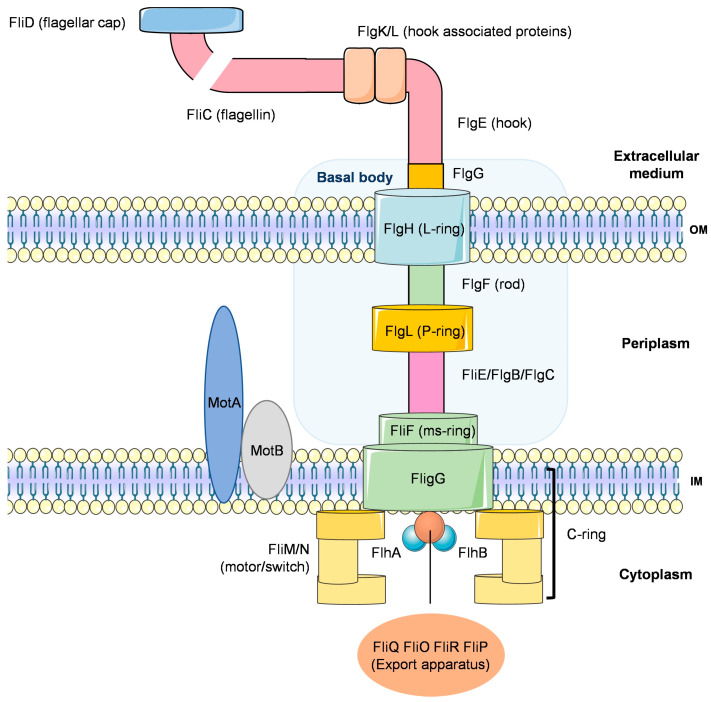
Schematic representation of a flagellum in *P. aeruginosa. P. aeruginosa* expresses a single polar flagellum. It is composed of a basal body: a L-ring connected with the LPS layer, a P-ring associated with the peptidoglycan layer, the MS-ring is located on the plasma membrane. The basal body is associated to a motor/switch, responsible for generating the rotation of the filament. The outer part of the flagellum is composed of a hook (protein FlgE), and a filament (protein FliC). The filament ends with capping (protein FliD).

*P. aeruginosa* has a single polar flagellum, composed of a flagellin protein named FliC [3]. The flagellum consists of a basal body and a motor situated within the cell membrane, and of a rod which extends through the peptidoglycan and outer layers of the cell, to which the hook and filament are attached (Figure 4). Swimming and swarming actions are driven by rotation of the flagellum, which allows *P. aeruginosa* to move. Forward propulsion results from an anti-clockwise rotation of the flagellum, while tumbling motion results from its clockwise revolution. Changes in the direction of movement result from chemotaxis and are triggered by chemical signals [100,102].

Twitching motility involves the movement of cell surface appendages called pili. In *P. aeruginosa*, type IV pili (T4P) are the specific pili. These pili are synthesized within the cell and this process involves a series of proteins (PilA, PilC, PilB, PilQ) that intervene in the rapid assembly and disassembly, leading to a twitching movement [103]. This movement results in small clusters of cells that come together to form a networked cell structure, with the vanguard actively involved in migration, fixation, and biofilm formation [100,104]. The nucleotide signaling molecule cyclic-di-GMP controls sessile motility switch. The cyclic-di-GMP regulates a transition from the mobile and planktonic state to sessile, favoring biofilm development, by inhibiting the flagella and pili that allow for this type of movement. When levels of this nucleotide signaling molecule are low, a motile lifestyle is favored [103]. Badal and his collaborators show that some of the two-component systems biofilm regulators regulate flagellum-dependent swimming motility and type IV pilus-dependent contraction motility. For example, when comparing the two-component system mutants with their isogenic parent *P. aeruginosa* PA14 (using controls PA14*rhlR* as positive control and PA14*pilW* and negative control), it was demonstrated that parent PA14 and PA14*rhlR* were capable of twitching but not the PA14*pilW* mutant [105].Thus, motility is a virulence factor that is essential for several functions, such as surface detection and translocation, lifestyle change, and biofilm formation. Motility and adherence seem to play important roles in triggering host immune responses, biofilm formation, and the development of chronic infection [3].

#### 4.1.3. Secondary Metabolites

*P. aeruginosa* uses peptides and proteins, especially a series of secondary metabolites that give them an advantage in different environments, as virulence factors. These metabolites negatively affect prokaryotic and eukaryotic cells, through growth inhibition or cell death [106]. Within the secondary metabolites, the most important classes for virulence are phenazine, alkyl quinolones, homoserine lactones, and rhamnolipids [107]. Phenazines, such as pyocyanin, are one of the most important secondary metabolites of *P. aeruginosa*. They are redox-active, colored heterocyclic compounds and are responsible for the green fluorescence of the *P. aeruginosa* [108]. Basically, pyocyanin can cause harmful oxidative stress to host cells by generating reactive oxygen species, superoxide, and hydrogen peroxide [109,110]. The genes required to produce pyocyanin are two single genes, *phzM* and *phzS* (responsible for the final conversion into pyocyanin) and two seven-gene homologous operons, phzA1B1C1D1E1F1G1 and phzA2B2C2D2E2F2G2 [111]. The operons encode the synthesis of phenazine-1-carboxylic acid from its precursor, chorismic acid. Phenazine-1-carboxylic acid is then converted to pyocyanin by a methyltransferase encoded by the *phzM* gene and a flavin-containing monooxygenase encoded by the *phzS* gene [112].

*P. aeruginosa* is one of the few organisms capable of synthesizing hydrogen cyanide (HCN) [113]. This secondary metabolite is maximally produced under high cell densities and at low oxygen tension during the transition from exponential to stationary growth phase. The HCN synthase is encoded in an operon, *hcnABC* [114]. The CN- ions are non-competitive inhibitors, as they can bind to Fe^3+^ in heme, which in turn binds to cytochrome c oxidase, consequently preventing oxygen from binding. Thus, the host cell is unable to perform aerobic respiration [115]. Among bacteria, HCN has been shown to kill competitors by increasing their susceptibility to antibiotics, through inhibition of efflux pumps that depend on cytochrome oxidase activity [115,116].

#### 4.1.4. Multiple Signalling Systems in the Regulation of Virulence Genes

##### Quorum Sensing System

Bacterial communication is the term given to the regulation of bacterial behavior in response to the presence of other cells. Cell-to-cell communication is based on the generation and release of small signaling molecules. These small signaling molecules, called autoinducers, are involved in a form of regulation known as quorum sensing (QS) [117]. QS is often referred to as “density-dependent” because a critical density of bacterial cells is necessary for the level of autoinducer to reach the limit, stimulus concentration and bind to the activator protein, causing expression of the target gene [118]. QS signaling molecules also regulate the expression of various virulence factors, adhesion, and motility, and control biofilm formation by *P. aeruginosa* [119]. Three distinct quorum sensing systems have been described in *P. aeruginosa*: two LuxIR homologs (*las* and *rhl*), involving two N-acyl homoserine lactone (AHLs) dependent systems, and the 2-heptyl-3-hydroxy-4-(1H)-quinolone (PQS) [120,121].

The *las* system is composed of LasI, a synthase protein, which produces the autoinducer N-3-oxo-dodecanoyl homoserine lactone (3OC12HSL), that binds to the transcriptional activator protein (LasR) at a threshold concentration. The 3OC12HSL-LasR complex binds to promoter regions upstream of genes that encode for a range of virulence factors (such as elastase, exotoxin A, and alkaline protease). The *las* system positively regulates the *rhl* and *pqs* systems. On the other hand, the *rhl* system negatively regulates the *pqs* system. The *pqs* system can autoinducer PQS synthesis and activates over-expression of the *rhl* system [3]. When the induction of the *rhl* transcriptional activator protein, RhlR, binding with N-butyryl homoserine lactone, (C4HSL) occurs, it leads to the synthesis of the autoinducer synthase protein (RhlI) [122]. The Rh1R-C4HSL complex can also induce the expression of several genes encoding virulence factors (such as alkaline protease, elastase, rhamnolipid, pyocyanin, and HCN) and can activate genes under the control of LasR [123]. The *las* and *rhl* systems do not act independently of each other: rather, they are organized in an established hierarchy with a signaling cascade [124].

The third *P. aeruginosa* QS system, PQS, is synthesized via condensation of anthranilate and *β*-keto dodecanoate [125]. The *pqsABCD* operon encoded 2-heptyl-4(1H)-quinolone, PQS precursor, is converted to PQS by PqsH, a putative monooxygenase [117,126]. The LysR-type regulator PqsR (also known as MvfR) regulates the PQS. This system controls the biosynthesis of AHL-associated virulence factors like pyocyanin and rhamnolipids. It also affects biofilm development [127]. Thus, the PQS pathway is linked to the LasI/LasR and RhlI/RhlR QS systems, influencing the production of virulence factors. The PQS HHQ precursor also acts as a cell-to-cell signal. HHQ can be released into the extracellular medium and then be absorbed by neighbouring cells, where it can be converted to PQS by PqsH, or bind directly to PqsR, in both cases activating PQS-regulated gene expression to levels similar to those observed in response to PQS itself [107].

Specific environmental conditions and host immune factors were associated with changes in the QS response of *P. aeruginosa*. For example, under anaerobic conditions, QS systems and transcriptional regulators (such as ANR) allow for bacterial growth and expression of virulence [128].

##### GacS/GacA, Two-Component Systems

It should be noted that, besides the existence of the QS-systems, *P. aeruginosa* also has two-component regulatory systems that control both its virulence phenotype and its response to environmental stimuli. Basically, the two-component regulatory systems are formed by two proteins: a histidine kinase (HK) and a response regulator (RR). One of the most important of such systems is GacS/GacA, which plays a critical role in controlling *P. aeruginosa* infectious processes by counter-regulating the expression of virulence factors associated with acute infection, such as T3SS, type IV pili, and flagellum formation, as well as mechanisms associated with chronic infection, such as T6SS and biofilm formation [3]. The GacS/GacA system consists of a transmembrane hybrid sensor HK, GacS. The function of GacS is to phosphorylate the response regulator (RR) and histidine phosphotransfer (Hpt) domains, resulting in their transfer to GacA in the cytosol. GacA promotes the transcription of two regulatory sRNAs (RsmZ and RsmY). These two bind to the small RNA-binding protein RsmA, through characteristic GGA motifs, preventing it from attaching to its target mRNAs, avoiding their translation suppression and increasing mRNA turnover, positively regulating the expression of genes related to T3SS, type IV pili, and flagellum formation, while negatively regulating the expression genes involved in T6SS and biofilm formation [129,130]. The GacS/GacA regulators are LadS, RetS, and PA1611. LadS, a hybrid histidine kinase, promotes autophosphorylation of GacS by shuttling additional phosphate groups into its Hpt domain [131]. RetS, a hybrid histidine kinase, captures phosphate groups from the GacS, preventing the autophosphorylation of GacS [132]. The PA1611, a hybrid histidine kinase, interacts with RetS, thus preventing RetS from inhibiting GacS [133]. However, the ligands binding to the sensor domain of GacS are unknown. Studies in mice have shown that the loss of RsmA results in a reduction in colonization during the early stages of acute infection, but also favors chronic infection, resulting in increased inflammation in the lungs of infected mice [129].

##### Nucleotide Signals

Studies related to signal propagation have shown that a group of nucleotide-based molecules also plays an important role in bacterial control [107]. The 3′, 5′-cyclic adenosine monophosphate (cAMP) is an essential signaling molecule for the regulation of virulence factors in acute infections [134]. Basically, cAMP binds and activates transcription factors from the CRP family. In the genome of *P. aeruginosa* PAO1, cAMP synthesis is driven by two adenylate cyclases, CyaA and CyaB [135,136]. In mice, the enzymatic activity of CyaB is increased in response to specific signals, such as low calcium levels, showing that the most significant contribution to cellular levels of cAMPs is derived from CyaB [137]. The production of various virulence factors such as upregulating exotoxin A, T3SS, T2SS, and the *las* and *rhl* QS systems, can be directly or indirectly controlled by binding cAMP to the homologous CRP VfR regulator (virulence factor regulator). On the other hand, flagellar gene expression is downregulated by repressing expression of *fleQ* [138,139]. In *P. aeruginosa*, mutations in *mucA* (virulence factor that regulates alginate) and activation of AlgU inhibit cAMP-VfR signaling, proving that this type of cAMP-Vfr signaling works as a complex signalling cascade with many regulatory inputs [107].

The other group is the cellular alarmones ppGpp (5′-diphosphate 3′-diphosphate guanosine) and pppGpp (5′-triphosphate 3′-diphosphate guanosine) [140]. The cellular pool of ppGpp can be controlled by two classes of enzymes: monofunctional synthetases and bifunctional synthetase–hydrolase enzymes. The monofunctional synthetases (RelA proteins) use GTP and ATP to generate pppGpp, which is then converted in ppGpp. Bifunctional synthetase–hydrolase enzymes (SpoT and Rel proteins) can act on ppGpp and pppGpp, and ppGpp hydrolyses the nucleotides to yield either GDP and pyrophosphate (PP_i_), whereas pppGpp hydrolyses GTP and PP_i_ [141,142]. The association of ppGpp with the transcriptional regulator DksA (global regulator of metabolism) allows the interaction between ppGpp and the RNA polymerase, inhibiting the transcription of ribosomal RNA promoters. This inhibition occurs simultaneously with the activation of stress response genes transcription and upregulation of amino acid biosynthesis pathways [140,143]. The interaction of ppGpp and DksA can also indirectly regulates the transcription process known as σ-factor competition. *P. aeruginosa* has many σ-factors encoded in its genome, with 24 putative σ-factors identified so far [144]. In general, the σ-factor binds to the RNA polymerase core enzyme producing a holoenzyme. Once this happens, the σ-factor can recognize its cognate promoter sequence leading to a binding process between them. This facilitates the opening of the double-stranded DNA, leading to the initiation of transcription by allowing the transcription machinery to bind. In the end, elongation of the transcript from the template strand leads to the finished mRNA that can be then translated into proteins or processed further [144,145]. σ-factors are responsible for the transcription of genes that are necessary under certain conditions. For example, σ-factor σ^70^ (also known as RpoD), is needed in exponential growth while σ^38^ (also known as RpoS), is needed in the stationary phase [143,144]. Additionally, the expression of RpoS is important for tolerance against antibiotics during stationary phase [146].

The cyclic-3′5′-diguanylic acid (c-di-GMP) is another nucleotide-signaling molecule, playing a very important role in the post-transcriptional regulation of biofilm formation. This small molecule has the ability to increase the triggers for biofilm formation, meanwhile inhibiting motility [147]. The c-di-GMP signaling system is very complex and consists of two groups of proteins: diguanylate cyclases (DGCs) (containing GGDEF motif), responsible for c-di-GMP synthesis, and phosphodiesterase (PDEs) (containing EAL or HD-GYP motif) involved in c-di-GMP degradation [3]. These enzymes control many factors, such as biosynthesis of adhesins and exopolysaccharides, motility, environmental stress adaptation, synthesis of secondary metabolites, virulence in animal and plant hosts, among others [148]. The transduction of phosphorylation events via the GacS/GacA regulatory systems controls biofilm formation. In this GacS/GacA two-component system, the RsmA RNA-binding protein negatively controls biofilm formation pathways, by inhibiting production of GGDEF/EAL encoding proteins and it consequently inhibits the increase in cyclic di-GMP levels. When *P. aeruginosa* is under stress, this pathway generates RsmY and RsmZ (non-coding RNAs), which neutralize the translational repression activity of RsmA. Accordingly, there is an increase in the level of cyclic di-GMP, promoting the biosynthesis of polysaccharides (alginate and Pel) and resulting in biofilm formation [107].

Therefore, quorum sensing and the multiple different bacterial signaling systems have a major impact on bacterial control in relation to the environment and in the regulation of its various virulence factors. A better understanding of these multiple signaling systems will, in the future, allow for a better control and combat of bacterial infections.

### 4.2. Biofilm

Bacterial biofilms are structured colonial aggregates of cells, most often embedded in a matrix of extracellular polymeric substances (EPS) [149]. These biofilms are resistant to antibiotics, phagocytosis, and surfactants, making them difficult to remove once established [150]. Biofilm formation in *P. aeruginosa* is triggered upon association with a biotic or abiotic surface, leading to the specific production and secretion of extracellular matrix, that can include exopolysaccharides, DNA and/or protein [151]. The development of *P. aeruginosa* PAO1 biofilms is divided in five stages. The first stage is the reversible attachment, where planktonic cells attach to the substratum by means of their flagella. In the second stage, there is a continuous influx of motile cells to the site of biofilm initiation. Subsequently, the bacteria form microcolonies. At this stage, type IV pili maintain twitching motility. After definitive fixation, *P. aeruginosa* starts the expression of the *algC*, *algD*, and *algU* genes, responsible for the synthesis of the alginate extracellular polymeric substances. At the third stage, cell proliferation and loss of motility organelles mark the beginning of the biofilm maturation process, during which exponential upregulation of certain genes is observed. In the fourth stage, biofilm maturation and thickening are now complete. The initial monolayer cell mat transforms into a three-dimensional structure and structurally adopts a mushroom-shaped multicellular assembly. The fifth and last state is the detachment of single cells or aggregates from the biofilm, leading to the dispersion of the planktonic cells back into the environment, which can re-form a new biofilm elsewhere. For dispersion and separation of bacterial cells from the biofilm, the swimming capacity of *P. aeruginosa* and enzymatic degradation of the EPS matrix are important factors [151,152].

In *P. aeruginosa*, the *Psl* system, encoded by the *psl* operon, which contains many *psl* genes, contributes to the formation of biofilm, allowing to increase cell surface and intercellular adhesion [153]. The *psl* locus encodes the synthesis of polysaccharides rich in galactose and mannose, which play an essential role in the cell surface, cell interactions, and biofilm formation. Pellicle (Pel) polysaccharide, a glucose-rich matrix material, is another biofilm formation system. It is encoded by the *pelABCDEF* operon, containing six genes. When this operon is active, a glucose-rich, cellulase-sensitive matrix is created [154,155]. The Pel and Psl systems are involved in the early stages of biofilm formation [152]. The *pel* operon is necessary for the formation of biofilm on certain surfaces. It was demonstrated when comparing *P. aeruginosa* strain PA14 and *pelA* mutant of PA14*pelA*::Tn that the increase in biomass was significantly greater in wild type PA14 than in PA14*pelA* at all time points after inoculation [105].

**Figure 5:**
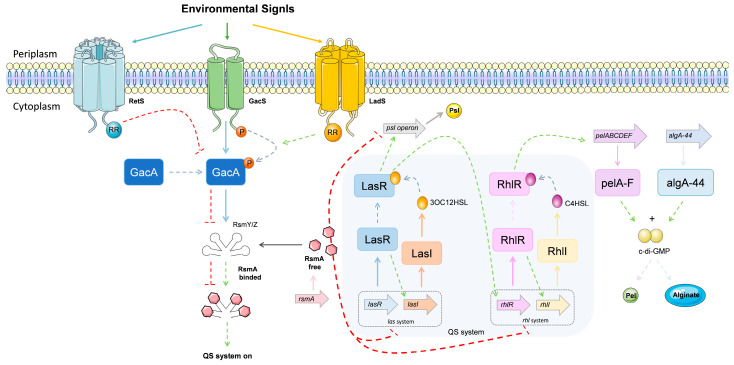
Bacterial systems and factors implicated in biofilm formation by *P. aeruginosa*. All the three systems are involved in biofilm formation: quorum sensing system (QS), two-component regulatory system GacS/GacA and RetS/LadS and the nucleotide signalling molecule c-di-GMP. The P represents phosphorylation, and the RR represents response regulator domain receiver. In the two-component regulatory system, upon autophosphorylation of GacS, transfers a phosphate group to GacA, through LadS (hybrid histidine kinase). The GacA upregulates the expression of the small regulatory RNAs (*RsmY/Z*) that will capture RNA-binding protein RsmA, a repressor that post-transcriptionally regulates the *psl* operon. The interaction between GacS and RetS makes phosphorylation of GacS repress. In QS, in las system, the *LasR* gene is transcribed giving rise to the LasR protein that binds to the promoter of the *psl* operon. The las system positively regulates the *rhl* system. The RhIR (encoded by *RhlR* gene which is in the rhl system) promotes the transcription of *pelABCDEF*. The binding of c-di-GMP to the protein’s pelA-F and Alg44 is necessary for the formation of polymer Pel and alginate, respectively.

Regulation of biofilm formation involves multiple bacterial machineries, such as the QS systems (*las* and *rhl*), two-component regulatory systems, and the nucleotide signaling molecule c-di-GMP (Figure 5) [127,129,147]. The *las* system intervenes in biofilm formation and maturation. The QS regulator LasR appears to bind to the promoter region of the *psl* operon, suggesting that this QS system regulates *psl* expression [156]. On the other hand, the *rhl* system intervenes in the formation of biofilm by increasing biosynthesis of the Pel polysaccharide. For instance, the transcription of the *pelABCDEF* operon has been reported to be drastically reduced in the *RhlI* mutant, however, the *rhlR* mutant did not [105,157]. In two-component regulatory systems (GacS/GacA), the RsmA represses the expression of the *pel* and *psl* genes. The interaction between GacS and RetS makes phosphorylation of GacS impossible and, consequently, there is no inhibition of RsmA, making RetS a negative regulator for biofilm formation [130]. The binding of c-di-GMP and proteins PelD and AlgA-44 is necessary for formation of the Pel polymer and alginate, respectively. Thus, high levels of c-di-GMP promote polysaccharide biosynthesis, while low levels promote motility through flagellar formation [147].

eDNA is also a major matrix component of *P. aeruginosa* biofilm. It participates in the early development of biofilms and facilitates its motility-mediated expansion. Furthermore, biofilms with an eDNA deficit are more sensitive to detergents [152]. 

### 4.3. Antibiotic Resistance

The use of antibiotics to treat infectious diseases has proven clinically successful, although researchers say that success comes at the expense of resistance. There are several reasons for acquiring the various types of resistance presently encountered. The direct cause of drug resistance is exposure of microorganisms to antibiotics, which leads to the natural selection of individuals with resistance genes to various antibiotics [158]. The plasticity of the bacterial genome, through spontaneous or induced mutations, also represents one of the main factors for the acquisition of antibiotic resistance. The horizontal transfer of genes or transmissible plasmids are also responsible for the acquisition of resistance genes among bacteria [159]. Therefore, recently, several studies have been conducted to develop new strategies to combat multidrug resistance pathogens. For example, Galdino and collaborators demonstrated in their studies that two metal complexes, Ag-phendione and Cu-phendione, as powerful inhibitors of lasB, since these complexes are able to interact with the active site of lasB and decrease the activity of purified lasB and from the secreted lasB. These complexes can be a promising avenue for the treatment of antibiotic-resistant *P. aeruginosa* through the lasB target, since this enzyme promotes several pathophysiological events during bacterial-host interactions [160]. Metal-based antibacterial complexes have been studied as an alternative to conventional antibiotics due to their differentiating modes of action and effective in combating pathogens [161]. Namely, silver, gold, bismuth, ruthenium, copper and manganese are the most studied compounds whose effects show a promising impact in combating antimicrobial resistance. These metal-based antibacterial complexes act essentially at the level of the DNA, RNA and bacterial membrane, disrupting the basic survival functions of the bacteria [161,162]. In addition to these more modern alternatives, the use of phages and bacteriocins has also been studied as an alternative to antibiotics [163,164].

*P. aeruginosa* infections are increasingly difficult to treat, as this pathogen has a natural resistance to multiple antibiotics, and the number of its multidrug-resistant strains is increasing globally [165]. Many antibiotics are used to treat *P. aeruginosa* infections, including a number of β-lactams such as cephalosporins (e.g., ceftazidime), carbapenems (e.g., meropenem, imipenem), and monobactams (e.g., aztreonam), aminoglycosides (e.g., gentamicin, tobramycin, amikacin), fluoroquinolones (ciprofloxacin, levofloxacin), and, recently, polymyxins (e.g., colistin, polymyxin B). Normally, the major mechanisms used to counter antibiotic attack can be classified into intrinsic, acquired, and adaptive resistance [151].

#### 4.3.1. Intrinsic Antibiotic Resistance

The intrinsic antibiotic resistance means the innate ability to decrease the effectiveness of a particular antibiotic through structural or functional characteristics conferring protection against toxic molecules and antimicrobials [166]. *P. aeruginosa* exhibits lower outer membrane permeability compared to other Gram-negative bacteria. The Gram-negative membrane is an asymmetric bilayer of phospholipid and LPS, and it works as a selective barrier to prevent the penetration of antibiotics [167]. To survive, *P. aeruginosa* must allow the entry of nutrients. This entry is accomplished through a collection of β-barrel proteins producing water-filled diffusion channels called porins [168]. The family of porins can be divided into four classes: non-specific porins, specific porins, gated porins, and efflux porins. Non-specific porins, the main of which is OprF, allow the slow diffusion of most small hydrophilic molecules. Specific porins, OprB, OprD, OprE, OprO, and OprP, have specific sites that bind to a particular set of molecules. Gated porins, TonB-dependent, OprC, and OprH, are responsible for the uptake of ion complexes. The OprM, OprN, and OprJ efflux porins are important components of efflux pumps [169]. Porins not only play an important role in the transport of sugars, amino acids and other nutrients, but are also involved in the transport of certain hydrophilic antibiotics such as β-lactams, aminoglycosides, tetracyclines, and some fluoroquinolones [168]. In *P. aeruginosa*, one of the intrinsic mechanisms is the limitation of entry of hydrophilic antibiotics, through decreased outer membrane permeability, by reducing the number of non-specific porin proteins and replacing the porins with specific or selective channels that absorb only the necessary nutrients [170]. The other intrinsic mechanisms occur in carbapenem-resistant clinical strains of *P. aeruginosa*, which have been shown to be deficient in the OprD porin.

Efflux pumps also play an important role in intrinsic antibiotic resistance. These efflux pumps can be substrate-specific or can extrude a wide range of compounds, including antibiotics from several structurally unrelated classes [171]. Efflux pumps have been classified in five superfamilies, namely: ATP-binding cassette, small multidrug resistance, major facilitator, resistance-nodulation-division (RND), and multidrug and toxic compound extrusion. PAO1 genome encodes multiple efflux pumps from all five superfamilies, but the resistance-nodulation-division (RND) type is the most prevalent [172,173]. The four most important RND efflux pumps are MexAB-OprM, MexCD-OprJ, MexEF-OprN, and MexXY/OprM(OprA). There are other forms of multidrug efflux pumps, but they are not expressed in wild-type strains, albeit they may contribute to adaptive resistance such as MexJK, MexGHI-OpmD, MexVW, MexPQ-OpmE, MexMN, and TriABC [174]. It was demonstrated that the MexEF-OprN and MexGHI-OpmD sets can modulate QS systems by exporting the quinolone signalling molecule PQS, reducing its concentration and, consequently, reducing the production of the virulence factors, which favours the establishment of chronic infections [175]. The pumps consist of three components: an efflux transporter in the inner membrane, an outer membrane channel, and an accessory protein connecting the two in the periplasm. In this complex, the inner membrane protein captures the substrates from either the phospholipid bilayer in the inner membrane of the bacterial cell envelope or the cytoplasm and transports them into the extracellular medium via the outer membrane protein. The cooperation between these proteins is mediated by a periplasmic protein [173].

Another intrinsic resistance determinant in *P. aeruginosa* is the inducible *ampC* gene, encoding the hydrolytic enzyme β-lactamases [176]. β-lactamases can be divided into four classes, on the basis of their amino acid sequences: A, B, C, and D. Class B β-lactamases are metalloenzymes that require divalent zinc ions for β-lactam hydrolysis. On the other hand, classes A, C, and D hydrolyse β-lactams through an active site serine [177]. The expression of β-lactamase AmpC confers a low level of resistance to aminopenicillins and most cephalosporins strongly induce the production of AmpC, which consequently hydrolyses these substrates [174]. In wild-type basal expression of *ampC*, during normal cell wall recycling, 1,6-anhydromuropeptides are removed from the cell wall and transported into the cytoplasm via the AmpG permease. The 1,6-anhydromuropeptides are cleaved by AmpD to generate free tripeptides, which are later converted into UDP-MurNAc-pentapeptides. This UDPMurNAc-pentapeptide interacts with AmpR bound to the *ampR-ampC* intergenic region, creating a conformation that represses transcription of *ampC*, resulting in lower production of AmpC [174]. In the presence of a β-lactam antibiotic, it enters the periplasmic space through the outer membrane porins and interacts with specific PBPs. Because of the increase in 1.6-anhydromuropeptides pools, AmpD is unable to efficiently process the higher levels of those fragments. Anhydro-MurNAc peptides replace UDP-MurNAc-pentapeptides and then bound to AmpR, resulting in a conformational change in the protein. AmpR is converted to a transcriptional activator, *ampC* is expressed at higher levels leading to the increase of AmpC in the periplasmic space [174,178]. AmpC, being a β-lactamase, interacts with the antibiotic leading to its degradation. *P. aeruginosa*, through the acquisition of ESBL genes, can also produce extended-spectrum-β-lactamases (ESBLs)which confer a high degree of resistance to the majority of β-lactam antibiotics, including penicillins and cephalosporins [179].

Antimicrobial peptides (AMPs) are also promising models for the development of new antibiofilm drugs. To date, more than 3000 natural AMPs have been isolated and characterized have been isolated and characterized [180]. Natural and artificially synthesized AMPs can be a gold mine for the development of new antimicrobial agents [181]. Due to their mechanism of action, which depends on the permeabilization of bacterial membranes, AMPs exhibit numerous advantages such as: (1) broad-spectrum activity; (2) low tendency to induce resistance and (3) high potential to target metabolically dormant cells that are found at a high rate within microbial biofilms [182]. There are numerous AMPs that have already been studied for their antimicrobial activity. Example, HBD2 (human beta-defensin 2), in nanomolar concentrations, and regardless of its chiral status, significantly reduces biofilm formation in *P. aeruginosa* without affecting its metabolic activity. This reduction in biofilm formation adds importance to the role of HBD2 in innate host defense during the initial interaction between host and pathogen [183]. Another study performed with semi-synthetic peptide lin-SB056-1 and its dendrimeric derivative (lin-SB056-1)2-K demonstrated that lin-SB056-1 was effective in reducing *P. aeruginosa* biofilm formation and that the dimeric derivative (lin-SB056-1) 2-K demonstrated greater biofilm inhibitory activity compared to lin-SB056-1 [182]. Analogs of jelleine-1, another AMP, were synthesized for their study of antimicrobial functions and toxicity. However, jelleine-1 has been shown to have low antimicrobial potency against the gram-negative and gram-positive bacteria tested. In order to overcome this obstacle, an attempt was made to replace amino acids in order to make it more effective. It was shown that the introduction of Trp and Arg in the jelleine-1 sequence significantly increased its antimicrobial efficiency [181]. Although more studies are needed for this AMP, it is a potential candidate for the treatment of gram-negative bacterial infection.

It was recently understood that *P. aeruginosa* has the ability to alter its membrane under phosphorus stress conditions and, consequently, the fight against antibiotics such as polymyxin B is more effective. Basically, glycerophospholipids are the main lipids that form a membrane lipid bilayer in bacteria. Under phosphorus limiting conditions, *P. aeruginosa* produces surrogate glycolipids in order to replace phospholipids in response to this limitation. This lipid renovation pathway is strictly conserved in all *P. aeruginosa* strains. As a result of this membrane renewal, there is a trade-off in terms of antibiotic resistance. These membrane-added glycolipids can protect the bacteria from attacks by cationic antimicrobial peptides, highlighting a new mechanism of resistance to polymyxin B [184].

#### 4.3.2. Acquired Resistance

Although there is intrinsic resistance to certain antibiotics without prior exposure to antibiotics, acquired resistance is a consequence of antibiotic exposure, which leads to the selection of chromosomal genetic mutations or horizontal acquisition of genetic elements (plasmids, transposons, among others) [185]. For example, plasmids do not harbor essential genes for bacterial functioning under normal conditions, but contain genes that are advantageous for bacterial growth and multiplication under unfavorable conditions, such as the antibiotic resistance genes, heavy metal resistance, and virulence genes [186]. However, plasmid typing in *P. aeruginosa* is particularly complicated due to insertion, deletion, co-integration, and exchange events that determine a low phylogenetic agreement between plasmid core genes [187]. Although acquisition of resistance genes is not as common in *P. aeruginosa* as in many other bacteria, it has been shown to acquire plasmid-encoded carbapenemases (single metallo- β-lactamases) that can hydrolyse most β-lactams, including carbapenems [188]. Lopatkin and collaborators demonstrated that reducing antibiotic use alone is not enough to combat antibiotic resistance, as plasmids are transferred at high rates even in the absence of antibiotic selection [189]. Horizontal gene transfer occurs by transformation, transduction, and conjugation. It is an important means for bacteria to acquire several resistance genes from their counterparts, as is the case of acquisition of resistance to aminoglycoside, fluoroquinolones, and β-lactam resistance genes [190]. Resistance to colistin (polymyxin E), an antibiotic that belongs to the polymyxin family, can be plasmid-mediated through mobilization of the *mcr*-*1* gene [191].

Interference with antibiotic targets is also a common strategy that bacteria use to prevent the action of antibiotics. For instance, modification of quinolone target sites is one such resistance mechanism. Quinolones inhibit bacterial DNA replication by targeting DNA gyrase and topoisomerase IV. When mutations in the *gyrA-gyrB* and *parC-parE* genes (encoding DNA gyrase and topoisomerase IV, respectively) occur, there is a decrease in the binding affinity of the encoded proteins to quinolones, resulting in greater resistance to these antibiotics in *P. aeruginosa* [192].

In *P. aeruginosa*, one of the acquired resistance mechanisms is resistance to carbapenems, which involves mutations that lead to loss (by a mutation in the *oprD* gene itself or in one of its regulatory proteins, such as mexT) or reduction in the production of the OprD specific outer membrane porin channel [193]. Mutations in the *mexT* gene not only reduce OprD expression but also increase MexEF-OprN expression, leading to higher resistance to imipenem and antibiotics that are substrates for this pump [194].

Another well-studied mechanism of acquired resistance in *P. aeruginosa* is the mutations that cause overexpression of enzymes that inactivate antibiotics [192]. In clinical isolates of *P. aeruginosa*, AmpC overexpression was mostly associated *ampC* gene mutations, a gene encoding the *ampC* repressor. This led to the hyperproduction of β-lactamases and consequently, greatly increased resistance to cephalosporins [151,195].

#### 4.3.3. Adaptive Resistance

Unlike other types of resistance, adaptive resistance depends on growth circumstances that trigger regulatory events in the cell, and susceptibility usually reverses when inducing conditions are removed. Adaptive resistance is basically induced by environmental stresses and by the presence of specific antibiotics [151]. In *P. aeruginosa*, the best characterized adaptive resistance mechanism is biofilm formation, associated with the development of resistance to polymyxins, aminoglycoside, and cationic antimicrobial peptides [196]. It is already known that biofilm-producing bacteria are more resistant to antimicrobial agents and to the host’s immune response compared to other bacteria [197]. For example, eDNA, in addition to being a major component of the *P. aeruginosa* biofilm matrix, also has the ability to acidify the cellular environment, changing membrane permeability and causing structural changes in the lipid A of LPS, leading to different responses to antimicrobials like colistin or to other polymyxins [198].

#### 4.3.4. The Case of Multiple Colistin Response Mechanisms

Polymyxin is a peptide antibiotic composed of five chemically different compounds (polymyxins A-E). In clinical practice, only polymyxin B and polymyxin E (colistin) are used [199]. For the treatment of some infections, colistin alone is still an effective antibiotic [200]. This antibiotic is used in human medicine, but it has also been widely used in veterinary medicine for the treatment of various infections or in farm animal production, to aid their growth [191,201]. Since 2006 the use antibiotics as growth promoters is no longer allowed in the European Union [202].

Colistin is polycationic and has both hydrophilic and lipophilic moieties. It causes destabilization of the outer membrane, leading to pore formation, thus increasing membrane permeability [203]. Colistin, which is positively charged, binds through electrostatic interaction to the negatively charged phosphate groups of lipid A subunits present in the structure of lipopolysaccharide (LPS) [199]. The initial binding to an anionic LPS component displaces divalent calcium cations (Ca^2+^) and magnesium (Mg^2+^) in a competitive way, damaging the LPS three-dimensional structure. It is subsequently inserted at the hydrophobic terminal acyl fat chain, the external outer membrane (OM) monolayer is thus expanded, and membrane permeability is increased [204].

Colistin resistance mechanisms can be accounted for by many different mechanisms [199,205]. The transmissibility of a plasmid that contains the colistin resistance gene (*mcr-1*) is one of the resistance mechanisms adopted by *P. aeruginosa* [206]. The fast dissemination of this resistance gene may be due to the recurrent use of colistin in veterinary medicine, which has consequently accelerated the dissemination between animals and humans [207].

Overall, two main mechanisms of resistance to colistin in *P. aeruginosa* are chromosomal mutation and adaptation. Resistance caused by the chromosomal mutation is hereditary and is independent of the presence of antibiotics. One such example is the overexpression of efflux pumps (MexAB-OprM and MexXY-OprM), which are expressed in wild-type cells and contribute to the intrinsic resistance of several antibiotics [208].

**Figure 6:**
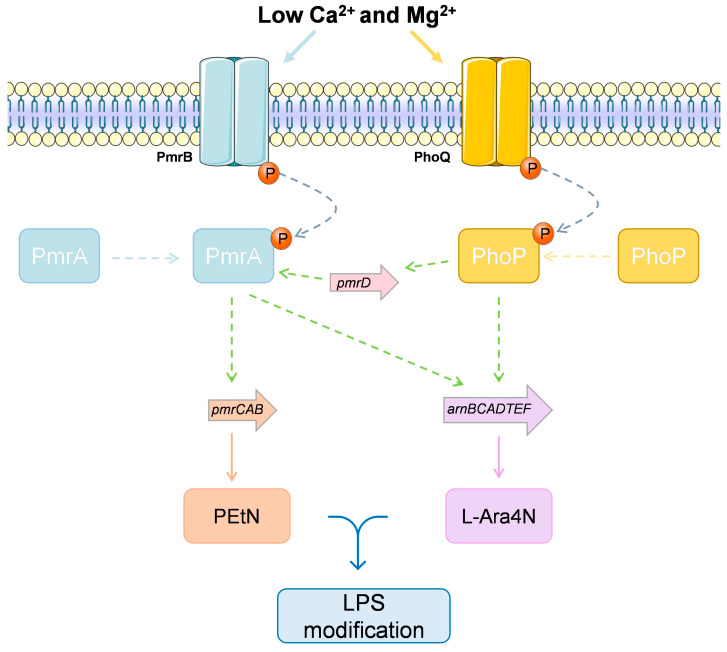
Colistin resistance mechanism by LPS modification in *P. aeruginosa*.

In *P. aeruginosa,* adaptive resistance to colistin occurs when extracellular concentrations of divalent cations Ca^2+^ and Mg^2+^ are limited and is controlled by the two-component regulators PmrA-PmrB and PhoP-PhoQ (Figure 6) [209,210]. The PhoP/PhoQ system is a global regulatory system that autoregulates the *oprH-phoP-phoQ* operon when there is a limitation of divalent cations in the environment. PhoP phosphorylation increases the transcription of several genes, such as *pmrD*, whose product binds to PmrA, when in the phosphorylated state [211]. In the PmrA/PmrB, the *pmrA/B* also directly controls the *arnBCADTEF* (also called *pmrHFIJKLM*) and *pmrCAB* operons, involved in LPS modification. The *pmrCAB* operon encodes PEtN and the *arnBCADTEF* encodes the enzymes for covalent addition of 4-amino-4-deoxy-L-arabinose (L-Ara4N) to lipid A. After binding to lipid A, changes in the negative charge of the cell membrane take place, due to neutralization of the negatively charged phospholipids [205,212,213]. Regardless of the characterization of these loci, the evolutionary dynamics of colistin resistance is still poorly understood and the adaptive mutations reported to date are probably incomplete [205].

## 5. Conclusions

This review clearly demonstrates the threat that *P. aeruginosa* represents to public health. Although many of the mechanisms and features of the metabolism of *P. aeruginosa* are still far from being fully understood, which constitutes an obstacle to build proper strategies to fight this problematic. It is true that the search for new antibiotics is at the heart of the solutions to achieve this, but researchers must also bear in mind that bacteria are, in general, fully armed and are adaptive organisms. Therefore, the problem should not be addressed only by the discovery of new antibiotics. That being said, all characteristics involving virulence and pathogenicity should also be addressed, taking into account the specific metabolic pathways that have an impact on the virulence of each organism. Secretion systems are proteins that allow the release of effector molecules, such as toxins, but also of proteins such as elastases, lipases, and proteases that can degrade molecules in the environment to release essential nutrients for the bacteria. In *P. aeruginosa*, biofilm formation and resistance to antimicrobial agents are important for the ability to establish infections and cause disease in humans and animals. All these mechanisms are regulated by multiple regulatory systems such as the quorum sensing system, two-component systems, and nucleotide signals. Understanding where and how these systems are regulated can provide essential information for the control of this pathogen.

## Data Availability

Not applicable.

## References

[1] World Health Organization (WHO) http://www.who.int/.

[2] De M Campos J.C., Antunes L.C., Ferreira R.B. (2020). Global priority pathogens: Virulence, antimicrobial resistance and prospective treatment options. Future Microbiol..

[3] Wu W., Jin Y., Bai F., Jin S. (2015). Pseudomonas aeruginosa. Molecular Medical Microbiology.

[4] Hauser A.R. (2009). The type III secretion system of *Pseudomonas aeruginosa:* Infection by injection. Nat. Rev. Microbiol..

[5] Frieri M., Kumar K., Boutin A. (2017). Antibiotic resistance. J. Infect. Public Health.

[6] Magiorakos A.P., Srinivasan A., Carey R.B., Carmeli Y., Falagas M.E., Giske C.G., Harbarth S., Hindler J.F., Kahlmeter G., Olsson-Liljequist B. (2012). Multidrug-resistant, extensively drug-resistant and pandrug-resistant bacteria: An international expert proposal for interim standard definitions for acquired resistance. Clin. Microbiol. Infect..

[7] Aloush V., Navon-Venezia S., Seigman-Igra Y., Cabili S., Carmeli Y. (2006). Multidrug-resistant *Pseudomonas aeruginosa:* Risk factors and clinical impact. Antimicrob Agents Chemother..

[8] Spiers A.J., Buckling A., Rainey P.B. (2000). The causes of Pseudomonas diversity. Microbiology.

[9] Migula W. (1900). System der Bakterien: Handbuch der Morphologie, Entwicklungsgeschichte und Systematik der Bakterien.

[10] Palleroni N.J. (2010). The Pseudomonas story. Environ. Microbiol..

[11] Ramos J.-L. (2011). Pseudomonas: Volume 1 Genomics, Life Style and Molecular Architecture.

[12] Pirnay J.P., Matthijs S., Colak H., Chablain P., Bilocq F., Van Eldere J., De Vos D., Zizi M., Triest L., Cornelis P. (2005). Global *Pseudomonas aeruginosa* biodiversity as reflected in a Belgian river. Environ. Microbiol..

[13] Mena K.D., Gerba C.P. (2009). Risk assessment of *Pseudomonas aeruginosa* in water. Rev. Environ. Contam. Toxicol..

[14] Remold S.K., Brown C.K., Farris J.E., Hundley T.C., Perpich J.A., Purdy M.E. (2011). Differential habitat use and niche partitioning by Pseudomonas species in human homes. Microb. Ecol..

[15] Crone S., Vives-Florez M., Kvich L., Saunders A.M., Malone M., Nicolaisen M.H., Martinez-Garcia E., Rojas-Acosta C., Catalina Gomez-Puerto M., Calum H. (2020). The environmental occurrence of *Pseudomonas aeruginosa*. APMIS.

[16] Khan H.A., Baig F.K., Mehboob R. (2017). Nosocomial infections: Epidemiology, prevention, control and surveillance. Asian Pac. J. Trop. Biomed..

[17] Abd H., Wretlind B., Saeed A., Idsund E., Hultenby K., Sandstrom G. (2008). *Pseudomonas aeruginosa* utilises its type III secretion system to kill the free-living amoeba Acanthamoeba castellanii. J. Eukaryot. Microbiol..

[18] Kung V.L., Ozer E.A., Hauser A.R. (2010). The accessory genome of *Pseudomonas aeruginosa*. Microbiol. Mol. Biol. Rev..

[19] Silveira M.C., Albano R.M., Asensi M.D., Carvalho-Assef A.P.D.A. (2016). Genetics; Evolution. Description of genomic islands associated to the multidrug-resistant *Pseudomonas aeruginosa* clone ST277. Infect. Genet. Evol..

[20] Battle S.E., Rello J., Hauser A.R. (2009). Genomic islands of *Pseudomonas aeruginosa*. FEMS Microbiol. Lett..

[21] Harrison E.M., Carter M.E., Luck S., Ou H.Y., He X., Deng Z., O’Callaghan C., Kadioglu A., Rajakumar K. (2010). Pathogenicity islands PAPI-1 and PAPI-2 contribute individually and synergistically to the virulence of *Pseudomonas aeruginosa* strain PA14. Infect. Immun..

[22] Granato E.T., Ziegenhain C., Marvig R.L., Kümmerli R. (2018). Low spatial structure and selection against secreted virulence factors attenuates pathogenicity in *Pseudomonas aeruginosa*. ISME J..

[23] Leone I., Chirillo M.G., Raso T., Zucca M., Savoia D. (2008). Phenotypic and genotypic characterization of *Pseudomonas aeruginosa* from cystic fibrosis patients. Eur. J. Clin. Microbiol. Infect. Dis..

[24] Palmer G.C., Whiteley M. (2015). Metabolism and Pathogenicity of *Pseudomonas aeruginosa* Infections in the Lungs of Individuals with Cystic Fibrosis. Microbiol. Spectr..

[25] Parkins M.D., Somayaji R., Waters V.J. (2018). Epidemiology, Biology, and Impact of Clonal *Pseudomonas aeruginosa* Infections in Cystic Fibrosis. Clin. Microbiol. Rev..

[26] Mesaros N., Nordmann P., Plesiat P., Roussel-Delvallez M., Van Eldere J., Glupczynski Y., Van Laethem Y., Jacobs F., Lebecque P., Malfroot A. (2007). *Pseudomonas aeruginosa:* Resistance and therapeutic options at the turn of the new millennium. Clin. Microbiol. Infect..

[27] Ferrara A.M. (2006). Potentially multidrug-resistant non-fermentative Gram-negative pathogens causing nosocomial pneumonia. Int. J. Antimicrob. Agents.

[28] Haenni M., Hocquet D., Ponsin C., Cholley P., Guyeux C., Madec J.Y., Bertrand X. (2015). Population structure and antimicrobial susceptibility of *Pseudomonas aeruginosa* from animal infections in France. BMC Vet. Res..

[29] Cabassi C.S., Sala A., Santospirito D., Alborali G.L., Carretto E., Ghibaudo G., Taddei S. (2017). Activity of AMP2041 against human and animal multidrug resistant *Pseudomonas aeruginosa* clinical isolates. Ann. Clin. Microbiol. Antimicrob..

[30] Hogardt M., Heesemann J. (2013). Microevolution of *Pseudomonas aeruginosa* to a chronic pathogen of the cystic fibrosis lung. Curr. Top. Microbiol. Immunol..

[31] Sommer L.M., Johansen H.K., Molin S. (2020). Antibiotic resistance in *Pseudomonas aeruginosa* and adaptation to complex dynamic environments. Microb. Genom..

[32] Diggle S.P., Whiteley M. (2020). Microbe Profile: *Pseudomonas aeruginosa:* Opportunistic pathogen and lab rat. Microbiology.

[33] Klockgether J., Cramer N., Wiehlmann L., Davenport C.F., Tummler B. (2011). *Pseudomonas aeruginosa* Genomic Structure and Diversity. Front. Microbiol..

[34] Stover C.K., Pham X.Q., Erwin A., Mizoguchi S., Warrener P., Hickey M., Brinkman F., Hufnagle W., Kowalik D., Lagrou M.J.N. (2000). Complete genome sequence of *Pseudomonas aeruginosa* PAO1, an opportunistic pathogen. Nature.

[35] Ozer E.A., Allen J.P., Hauser A.R. (2014). Characterization of the core and accessory genomes of *Pseudomonas aeruginosa* using bioinformatic tools Spine and AGEnt. BMC Genom..

[36] Chevalier S., Bouffartigues E., Bodilis J., Maillot O., Lesouhaitier O., Feuilloley M.G., Orange N., Dufour A., Cornelis P. (2017). Structure, function and regulation of *Pseudomonas aeruginosa* porins. FEMS Microbiol. Rev..

[37] Valot B., Guyeux C., Rolland J.Y., Mazouzi K., Bertrand X., Hocquet D. (2015). What It Takes to Be a *Pseudomonas aeruginosa* ? The Core Genome of the Opportunistic Pathogen Updated. PLoS ONE.

[38] Freschi L., Bertelli C., Jeukens J., Moore M.P., Kukavica-Ibrulj I., Emond-Rheault J.G., Hamel J., Fothergill J.L., Tucker N.P., McClean S. (2018). Genomic characterisation of an international *Pseudomonas aeruginosa* reference panel indicates that the two major groups draw upon distinct mobile gene pools. FEMS Microbiol. Lett..

[39] Freschi L., Vincent A.T., Jeukens J., Emond-Rheault J.G., Kukavica-Ibrulj I., Dupont M.J., Charette S.J., Boyle B., Levesque R.C. (2019). The *Pseudomonas aeruginosa* Pan-Genome Provides New Insights on Its Population Structure, Horizontal Gene Transfer, and Pathogenicity. Genome Biol. Evol..

[40] Mathee K., Narasimhan G., Valdes C., Qiu X., Matewish J.M., Koehrsen M., Rokas A., Yandava C.N., Engels R., Zeng E. (2008). Dynamics of *Pseudomonas aeruginosa* genome evolution. Proc. Natl. Acad. Sci. USA.

[41] Spencer D.H., Kas A., Smith E.E., Raymond C.K., Sims E.H., Hastings M., Burns J.L., Kaul R., Olson M.V. (2003). Whole-genome sequence variation among multiple isolates of *Pseudomonas aeruginosa*. J. Bacteriol..

[42] Wolfgang M.C., Kulasekara B.R., Liang X., Boyd D., Wu K., Yang Q., Miyada C.G., Lory S. (2003). Conservation of genome content and virulence determinants among clinical and environmental isolates of *Pseudomonas aeruginosa*. Proc. Natl. Acad. Sci. USA.

[43] Dobrindt U., Hochhut B., Hentschel U., Hacker J. (2004). Genomic islands in pathogenic and environmental microorganisms. Nat. Rev. Microbiol..

[44] Ho Sui S.J., Fedynak A., Hsiao W.W., Langille M.G., Brinkman F.S. (2009). The association of virulence factors with genomic islands. PLoS ONE.

[45] Sawa T., Momiyama K., Mihara T., Kainuma A., Kinoshita M., Moriyama K. (2020). Molecular epidemiology of clinically high-risk *Pseudomonas aeruginosa* strains: Practical overview. Microbiol. Immunol..

[46] Gaviard C., Jouenne T., Hardouin J. (2018). Proteomics of *Pseudomonas aeruginosa:* The increasing role of post-translational modifications. Expert Rev. Proteom..

[47] Darch S.E., McNally A., Harrison F., Corander J., Barr H.L., Paszkiewicz K., Holden S., Fogarty A., Crusz S.A., Diggle S.P. (2015). Recombination is a key driver of genomic and phenotypic diversity in a *Pseudomonas aeruginosa* population during cystic fibrosis infection. Sci. Rep..

[48] Goodman A.L., Merighi M., Hyodo M., Ventre I., Filloux A., Lory S.J.G. (2009). Direct interaction between sensor kinase proteins mediates acute and chronic disease phenotypes in a bacterial pathogen. Genes Dev..

[49] Desvaux M., Hébraud M., Talon R., Henderson I.R. (2009). Secretion and subcellular localizations of bacterial proteins: A semantic awareness issue. Trends Microbiol..

[50] Dettman J.R., Rodrigue N., Aaron S.D., Kassen R. (2013). Evolutionary genomics of epidemic and nonepidemic strains of *Pseudomonas aeruginosa*. Proc. Natl. Acad. Sci. USA.

[51] Sousa A.M., Pereira M.O. (2014). *Pseudomonas aeruginosa* Diversification during Infection Development in Cystic Fibrosis Lungs-A Review. Pathogens.

[52] Ma Q., Zhai Y., Schneider J.C., Ramseier T.M., Saier M.H. (2003). Protein secretion systems of *Pseudomonas aeruginosa* and P fluorescens. Biochim. Biophys. Acta.

[53] Bleves S., Viarre V., Salacha R., Michel G.P., Filloux A., Voulhoux R. (2010). Protein secretion systems in *Pseudomonas aeruginosa:* A wealth of pathogenic weapons. Int. J. Med. Microbiol..

[54] Delepelaire P. (2004). Type I secretion in gram-negative bacteria. Biochim. Biophys. Acta.

[55] Guzzo J., Pages J., Duong F., Lazdunski A., Murgier M. (1991). *Pseudomonas aeruginosa* alkaline protease: Evidence for secretion genes and study of secretion mechanism. J. Bacteriol..

[56] Matsumoto K. (2004). Role of bacterial proteases in pseudomonal and serratial keratitis. Biol. Chem..

[57] Costa T.R., Felisberto-Rodrigues C., Meir A., Prevost M.S., Redzej A., Trokter M., Waksman G. (2015). Secretion systems in Gram-negative bacteria: Structural and mechanistic insights. Nat. Rev. Microbiol..

[58] Cianciotto N.P. (2005). Type II secretion: A protein secretion system for all seasons. Trends Microbiol..

[59] Ball G., Durand É., Lazdunski A., Filloux A. (2002). A novel type II secretion system in *Pseudomonas aeruginosa*. Mol. Microbiol..

[60] Rybtke M., Berthelsen J., Yang L., Høiby N., Givskov M., Tolker-Nielsen T. (2015). The LapG protein plays a role in *Pseudomonas aeruginosa* biofilm formation by controlling the presence of the CdrA adhesin on the cell surface. Microbiologyopen.

[61] Casilag F., Lorenz A., Krueger J., Klawonn F., Weiss S., Häussler S. (2016). The LasB Elastase of *Pseudomonas aeruginosa* Acts in Concert with Alkaline Protease AprA to Prevent Flagellin-Mediated Immune Recognition. Infect. Immun..

[62] Korotkov K.V., Sandkvist M., Hol W.G.J. (2012). The type II secretion system: Biogenesis, molecular architecture and mechanism. Nat. Rev. Microbiol..

[63] Ostroff R.M., I Vasil A., Vasil M.L. (1990). Molecular comparison of a nonhemolytic and a hemolytic phospholipase C from *Pseudomonas aeruginosa*. J. Bacteriol..

[64] Allured V.S., Collier R.J., Carroll S.F., McKay D.B. (1986). Structure of exotoxin A of *Pseudomonas aeruginosa* at 3.0-Angstrom resolution. Proc. Natl. Acad. Sci. USA.

[65] Mapipa Q., Digban T.O., Nnolim N.E., Nwodo U.U. (2021). Antibiogram profile and virulence signatures of *Pseudomonas aeruginosa* isolates recovered from selected agrestic hospital effluents. Sci. Rep..

[66] Sawa T., Shimizu M., Moriyama K., Wiener-Kronish J.P. (2014). Association between *Pseudomonas aeruginosa* type III secretion, antibiotic resistance, and clinical outcome: A review. Crit. Care.

[67] Jia J., Alaoui-El-Azher M., Chow M., Chambers T.C., Baker H., Jin S. (2003). c-Jun NH 2 -Terminal Kinase-Mediated Signaling Is Essential for *Pseudomonas aeruginosa* ExoS-Induced Apoptosis. Infect. Immun..

[68] Rangel S.M., Logan L.K., Hauser A.R. (2014). The ADP-Ribosyltransferase Domain of the Effector Protein ExoS Inhibits Phagocytosis of *Pseudomonas aeruginosa* during Pneumonia. mBio.

[69] Rangel S.M., Diaz M.H., Knoten C.A., Zhang A., Hauser A.R. (2015). The Role of ExoS in Dissemination of *Pseudomonas aeruginosa* during Pneumonia. PLoS Pathog..

[70] Engel J., Balachandran P. (2009). Role of *Pseudomonas aeruginosa* type III effectors in disease. Curr. Opin. Microbiol..

[71] Urbanowski M.L., Lykken G.L., Yahr T. (2005). A secreted regulatory protein couples transcription to the secretory activity of the *Pseudomonas aeruginosa* type III secretion system. Proc. Natl. Acad. Sci. USA.

[72] McCaw M.L., Lykken G.L., Singh P.K., Yahr T.L. (2002). ExsD is a negative regulator of the *Pseudomonas aeruginosa* type III secretion regulon. Mol. Microbiol..

[73] Brutinel E.D., Vakulskas C.A., Brady K.M., Yahr T.L. (2008). Characterization of ExsA and of ExsA-dependent promoters required for expression of the *Pseudomonas aeruginosa* type III secretion system. Mol. Microbiol..

[74] Ahator S.D., Zhang L. (2019). Small Is Mighty—Chemical Communication Systems in *Pseudomonas aeruginosa*. Annu. Rev. Microbiol..

[75] Vakulskas C.A., Brady K.M., Yahr T.L. (2009). Mechanism of Transcriptional Activation by *Pseudomonas aeruginosa* ExsA. J. Bacteriol..

[76] Dasgupta N., Lykken G.L., Wolfgang M.C., Yahr T.L. (2004). A novel anti-anti-activator mechanism regulates expression of the *Pseudomonas aeruginosa* type III secretion system. Mol. Microbiol..

[77] Rietsch A., Vallet-Gely I., Dove S.L., Mekalanos J.J. (2005). ExsE, a secreted regulator of type III secretion genes in *Pseudomonas aeruginosa*. Proc. Natl. Acad. Sci. USA.

[78] Yahr T.L., Wolfgang M.C. (2006). Transcriptional regulation of the *Pseudomonas aeruginosa* type III secretion system. Mol. Microbiol..

[79] Zhou L., Wang J., Zhang L.-H. (2007). Modulation of Bacterial Type III Secretion System by a Spermidine Transporter Dependent Signaling Pathway. PLoS ONE.

[80] Iyer R., Williams C., Miller C. (2003). Arginine-Agmatine Antiporter in Extreme Acid Resistance in *Escherichia coli*. J. Bacteriol..

[81] Kwon D.H., Lu C.-D. (2006). Polyamines Induce Resistance to Cationic Peptide, Aminoglycoside, and Quinolone Antibiotics in *Pseudomonas aeruginosa* PAO1. Antimicrob. Agents Chemother..

[82] El-Halfawy O., Valvano M.A. (2015). Antimicrobial Heteroresistance: An Emerging Field in Need of Clarity. Clin. Microbiol. Rev..

[83] Kwon D.H., Lu C.-D. (2006). Polyamines Increase Antibiotic Susceptibility in *Pseudomonas aeruginosa*. Antimicrob. Agents Chemother..

[84] Filloux A. (2011). Protein Secretion Systems in *Pseudomonas aeruginosa:* An Essay on Diversity, Evolution, and Function. Front. Microbiol..

[85] Wilhelm S., Tommassen J., Jaeger K.-E. (1999). A Novel Lipolytic Enzyme Located in the Outer Membrane of *Pseudomonas aeruginosa*. J. Bacteriol..

[86] Wilhelm S., Gdynia A., Tielen P., Rosenau F., Jaeger K.-E. (2007). The Autotransporter Esterase EstA of *Pseudomonas aeruginosa* Is Required for Rhamnolipid Production, Cell Motility, and Biofilm Formation. J. Bacteriol..

[87] Hodak H., Jacob-Dubuisson F. (2007). Current challenges in autotransport and two-partner protein secretion pathways. Res. Microbiol..

[88] Salacha R., Kovačić F., Brochier-Armanet C., Wilhelm S., Tommassen J., Filloux A., Voulhoux R., Bleves S. (2010). The *Pseudomonas aeruginosa* patatin-like protein PlpD is the archetype of a novel Type V secretion system. Environ. Microbiol..

[89] Borlee B.R., Goldman A.D., Murakami K., Samudrala R., Wozniak D.J., Parsek M.R. (2010). *Pseudomonas aeruginosa* uses a cyclic-di-GMP-regulated adhesin to reinforce the biofilm extracellular matrix. Mol. Microbiol..

[90] Allsopp L.P., Wood T.E., Howard S.A., Maggiorelli F., Nolan L.M., Wettstadt S., Filloux A. (2017). RsmA and AmrZ orchestrate the assembly of all three type VI secretion systems in *Pseudomonas aeruginosa*. Proc. Natl. Acad. Sci. USA.

[91] Pukatzki S., Ma A., Sturtevant D., Krastins B., Sarracino D., Nelson W., Heidelberg J., Mekalanos J.J. (2006). Identification of a conserved bacterial protein secretion system in Vibrio cholerae using the Dictyostelium host model system. Proc. Natl. Acad. Sci. USA.

[92] Kanamaru S. (2009). Structural similarity of tailed phages and pathogenic bacterial secretion systems. Proc. Natl. Acad. Sci. USA.

[93] Bingle L.E., Bailey C.M., Pallen M. (2008). Type VI secretion: A beginner’s guide. Curr. Opin. Microbiol..

[94] Whitney J.C., Beck C.M., Goo Y.A., Russell A., Harding B.N., De Leon J.A., Cunningham D., Tran B.Q., Low D.A., Goodlett D.R. (2014). Genetically distinct pathways guide effector export through the type VI secretion system. Mol. Microbiol..

[95] Russell A., Hood R.D., Bui N.K., LeRoux M., Vollmer W., Mougous J.D. (2011). Type VI secretion delivers bacteriolytic effectors to target cells. Nature.

[96] Basler M., Pilhofer M., Henderson G.P., Jensen G.J., Mekalanos J.J. (2012). Type VI secretion requires a dynamic contractile phage tail-like structure. Nature.

[97] Lesic B., Starkey M., He J., Hazan R., Rahme L.G. (2009). Quorum sensing differentially regulates *Pseudomonas aeruginosa* type VI secretion locus I and homologous loci II and III, which are required for pathogenesis. Microbiology.

[98] Maurice N.M., Bedi B., Sadikot R.T. (2018). *Pseudomonas aeruginosa* Biofilms: Host Response and Clinical Implications in Lung Infections. Am. J. Respir. Cell Mol. Biol..

[99] O’May C., Tufenkji N. (2011). The Swarming Motility of *Pseudomonas aeruginosa* Is Blocked by Cranberry Proanthocyanidins and Other Tannin-Containing Materials. Appl. Environ. Microbiol..

[100] Harshey R.M. (2003). Bacterial Motility on a Surface: Many Ways to a Common Goal. Annu. Rev. Microbiol..

[101] Partridge J., Harshey R.M. (2012). Swarming: Flexible Roaming Plans. J. Bacteriol..

[102] Guttenplan S.B., Kearns D.B. (2013). Regulation of flagellar motility during biofilm formation. FEMS Microbiol. Rev..

[103] Burrows L.L. (2012). *Pseudomonas aeruginosa* Twitching Motility: Type IV Pili in Action. Annu. Rev. Microbiol..

[104] Mattick J.S. (2002). Type IV Pili and Twitching Motility. Annu. Rev. Microbiol..

[105] Badal D., Jayarani A.V., Kollaran M.A., Kumar A., Singh V. (2020). *Pseudomonas aeruginosa* biofilm formation on endotracheal tubes requires multiple two-component systems. J. Med. Microbiol..

[106] Strateva T., Mitov I. (2011). Contribution of an arsenal of virulence factors to pathogenesis of *Pseudomonas aeruginosa* infections. Ann. Microbiol..

[107] Nadal-Jimenez P., Koch G., Thompson J., Xavier K.B., Cool R.H., Quax W.J. (2012). The Multiple Signaling Systems Regulating Virulence in *Pseudomonas aeruginosa*. Microbiol. Mol. Biol. Rev..

[108] Managò A., Becker K.A., Carpinteiro A., Wilker B., Soddemann M., Seitz A.P., Edwards M.J., Grassmé H., Szabo I., Gulbins E. (2015). *Pseudomonas aeruginosa* Pyocyanin Induces Neutrophil DeathviaMitochondrial Reactive Oxygen Species and Mitochondrial Acid Sphingomyelinase. Antioxid. Redox Signal..

[109] Hassett D.J., Charniga L., Bean K., E Ohman D., Cohen M.S. (1992). Response of *Pseudomonas aeruginosa* to pyocyanin: Mechanisms of resistance, antioxidant defenses, and demonstration of a manganese-cofactored superoxide dismutase. Infect. Immun..

[110] Rada B., Leto T.L. (2009). Redox warfare between airway epithelial cells and Pseudomonas: Dual oxidase versus pyocyanin. Immunol. Res..

[111] Mavrodi D.V., Bonsall R.F., Delaney S.M., Soule M.J., Phillips G., Thomashow L.S. (2001). Functional Analysis of Genes for Biosynthesis of Pyocyanin and Phenazine-1-Carboxamide from *Pseudomonas aeruginosa* PAO1. J. Bacteriol..

[112] Huang L., Huang Y., Lou Y., Qian H., Xu D., Ma L., Jiang C., Zhang D. (2019). Pyocyanin-modifying genes phzM and phzS regulated the extracellular electron transfer in microbiologically-influenced corrosion of X80 carbon steel by *Pseudomonas aeruginosa*. Corros. Sci..

[113] Lenney W., Gilchrist F.J. (2011). *Pseudomonas aeruginosa* and cyanide production. Eur. Respir. J..

[114] Pessi G., Haas D. (2000). Transcriptional Control of the Hydrogen Cyanide Biosynthetic Genes hcnABC by the Anaerobic Regulator ANR and the Quorum-Sensing Regulators LasR and RhlR in *Pseudomonas aeruginosa*. J. Bacteriol..

[115] Cooper C.E., Brown G.C. (2008). The inhibition of mitochondrial cytochrome oxidase by the gases carbon monoxide, nitric oxide, hydrogen cyanide and hydrogen sulfide: Chemical mechanism and physiological significance. J. Bioenerg. Biomembr..

[116] Rijavec T., Lapanje A. (2016). Hydrogen Cyanide in the Rhizosphere: Not Suppressing Plant Pathogens, but Rather Regulating Availability of Phosphate. Front. Microbiol..

[117] Venturi V. (2006). Regulation of quorum sensing in *Pseudomonas*. FEMS Microbiol. Rev..

[118] Miller M.B., Bassler B.L. (2001). Quorum sensing in bacteria. Annu. Rev. Microbiol..

[119] Liu L., Li T., Cheng X.-J., Peng C.-T., Li C.-C., He L.-H., Ju S.-M., Wang N.-Y., Ye T.-H., Lian M. (2018). Structural and functional studies on *Pseudomonas aeruginosa* DspI: Implications for its role in DSF biosynthesis. Sci. Rep..

[120] Gallagher L.A., McKnight S.L., Kuznetsova M.S., Pesci E.C., Manoil C., Couture-Tosi E., Delacroix H., Mignot T., Mesnage S., Chami M. (2002). Functions Required for Extracellular Quinolone Signaling by *Pseudomonas aeruginosa*. J. Bacteriol..

[121] Kviatkovski I., Chernin L., Yarnitzky T., Frumin I., Sobel N., Helman Y. (2015). *Pseudomonas aeruginosa* activates the quorum sensing LuxR response regulator through secretion of 2-aminoacetophenone. Chem. Commun..

[122] Erickson D.L., Endersby R., Kirkham A., Stuber K., Vollman D.D., Rabin H.R., Mitchell I., Storey D.G. (2002). *Pseudomonas aeruginosa* Quorum-Sensing Systems May Control Virulence Factor Expression in the Lungs of Patients with Cystic Fibrosis. Infect. Immun..

[123] Bjarnsholt T., Jensen P., Jakobsen T.H., Phipps R., Nielsen A.K., Rybtke M.T., Tolker-Nielsen T., Givskov M., Høiby N., Ciofu O. (2010). Quorum Sensing and Virulence of *Pseudomonas aeruginosa* during Lung Infection of Cystic Fibrosis Patients. PLoS ONE.

[124] Pearson J.P., Pesci E., Iglewski B.H. (1997). Roles of *Pseudomonas aeruginosa* las and rhl quorum-sensing systems in control of elastase and rhamnolipid biosynthesis genes. J. Bacteriol..

[125] Bredenbruch F., Nimtz M., Wray V., Morr M., Müller R., Häussler S. (2005). Biosynthetic Pathway of *Pseudomonas aeruginosa* 4-Hydroxy-2-Alkylquinolines. J. Bacteriol..

[126] Déziel E., Lépine F., Milot S., He J., Mindrinos M.N., Tompkins R.G., Rahme L.G. (2004). Analysis of *Pseudomonas aeruginosa* 4-hydroxy-2-alkylquinolines (HAQs) reveals a role for 4-hydroxy-2-heptylquinoline in cell-to-cell communication. Proc. Natl. Acad. Sci. USA.

[127] De Kievit T.R., Gillis R., Marx S., Brown C., Iglewski B.H. (2001). Quorum-Sensing Genes in *Pseudomonas aeruginosa* Biofilms: Their Role and Expression Patterns. Appl. Environ. Microbiol..

[128] Lee J., Zhang L. (2014). The hierarchy quorum sensing network in *Pseudomonas aeruginosa*. Protein Cell.

[129] Mulcahy H., O’Callaghan J., O’Grady E.P., Maciá M.D., Borrell N., Gómez C., Casey P.G., Hill C., Adams C., Gahan C.G.M. (2008). *Pseudomonas aeruginosa* RsmA Plays an Important Role during Murine Infection by Influencing Colonization, Virulence, Persistence, and Pulmonary Inflammation. Infect. Immun..

[130] Goodman A.L., Kulasekara B., Rietsch A., Boyd D., Smith R.S., Lory S. (2004). A Signaling Network Reciprocally Regulates Genes Associated with Acute Infection and Chronic Persistence in *Pseudomonas aeruginosa*. Dev. Cell.

[131] Broder U.N., Jaeger T., Jenal U. (2016). LadS is a calcium-responsive kinase that induces acute-to-chronic virulence switch in *Pseudomonas aeruginosa*. Nat. Microbiol..

[132] Francis V.I., Waters E., Finton-James S.E., Gori A., Kadioglu A., Brown A.R., Porter S.L. (2018). Multiple communication mechanisms between sensor kinases are crucial for virulence in *Pseudomonas aeruginosa*. Nat. Commun..

[133] Bhagirath A.Y., Pydi S.P., Li Y., Lin C., Kong W., Chelikani P., Duan K. (2016). Characterization of the Direct Interaction between Hybrid Sensor Kinases PA1611 and RetS That Controls Biofilm Formation and the Type III Secretion System in *Pseudomonas aeruginosa*. ACS Infect. Dis..

[134] Inclan Y.F., Huseby M.J., Engel J.N. (2011). FimL Regulates cAMP Synthesis in *Pseudomonas aeruginosa*. PLoS ONE.

[135] Rietsch A., Mekalanos J.J. (2005). Metabolic regulation of type III secretion gene expression in *Pseudomonas aeruginosa*. Mol. Microbiol..

[136] Smith R.S., Wolfgang M.C., Lory S. (2004). An Adenylate Cyclase-Controlled Signaling Network Regulates *Pseudomonas aeruginosa* Virulence in a Mouse Model of Acute Pneumonia. Infect. Immun..

[137] Almblad H., Rybtke M., Hendiani S., Andersen J.B., Givskov M., Tolker-Nielsen T. (2019). High levels of cAMP inhibit *Pseudomonas aeruginosa* biofilm formation through reduction of the c-di-GMP content. Microbiology.

[138] Beatson S.A., Whitchurch C., Sargent J.L., Levesque R.C., Mattick J.S. (2002). Differential Regulation of Twitching Motility and Elastase Production by Vfr in *Pseudomonas aeruginosa*. J. Bacteriol..

[139] Wolfgang M.C., Lee V.T., Gilmore M.E., Lory S. (2003). Coordinate Regulation of Bacterial Virulence Genes by a Novel Adenylate Cyclase-Dependent Signaling Pathway. Dev. Cell.

[140] Potrykus K., Cashel M. (2008). (p)ppGpp: Still Magical?. Annu. Rev. Microbiol..

[141] Dalebroux Z.D., Svensson S.L., Gaynor E.C., Swanson M.S. (2010). ppGpp Conjures Bacterial Virulence. Microbiol. Mol. Biol. Rev..

[142] Amato S.M., Fazen C.H., Henry T.C., Mok W.W.K., Orman M.A., Sandvik E.L., Volzing K.G., Brynildsen M.P. (2014). The role of metabolism in bacterial persistence. Front. Microbiol..

[143] Dalebroux Z.D., Swanson M. (2012). ppGpp: Magic beyond RNA polymerase. Nat. Rev. Microbiol..

[144] Potvin E., Sanschagrin F., Levesque R.C. (2008). Sigma factors in *Pseudomonas aeruginosa*. FEMS Microbiol. Rev..

[145] Zenkin N., Severinov K. (2004). The role of RNA polymerase subunit in promoter-independent initiation of transcription. Proc. Natl. Acad. Sci. USA.

[146] Murakami K., Ono T., Viducic D., Kayama S., Mori M., Hirota K., Nemoto K., Miyake Y. (2005). Role forrpoSgene of *Pseudomonas aeruginosa* in antibiotic tolerance. FEMS Microbiol. Lett..

[147] Valentini M., Filloux A. (2016). Biofilms and Cyclic di-GMP (c-di-GMP) Signaling: Lessons from *Pseudomonas aeruginosa* and Other Bacteria. J. Biol. Chem..

[148] Ha D.-G., O’Toole G.A. (2015). c-di-GMP and its Effects on Biofilm Formation and Dispersion: A *Pseudomonas aeruginosa* Review. Microbiol. Spectr..

[149] Yang L., Hu Y., Liu Y., Zhang J., Ulstrup J., Molin S. (2011). Distinct roles of extracellular polymeric substances in *Pseudomonas aeruginosa* biofilm development. Environ. Microbiol..

[150] Høiby N., Ciofu O., Johansen H.K., Song Z., Moser C., Jensen P.Ø., Molin S., Givskov M., Tolker-Nielsen T., Bjarnsholt T. (2011). The clinical impact of bacterial biofilms. Int. J. Oral Sci..

[151] Taylor P.K., Yeung A.T., Hancock R.E. (2014). Antibiotic resistance in *Pseudomonas aeruginosa* biofilms: Towards the development of novel anti-biofilm therapies. J. Biotechnol..

[152] Rasamiravaka T., Labtani Q., Duez P., El Jaziri M. (2015). The Formation of Biofilms by *Pseudomonas aeruginosa:* A Review of the Natural and Synthetic Compounds Interfering with Control Mechanisms. BioMed Res. Int..

[153] Ma L., Jackson K.D., Landry R.M., Parsek M.R., Wozniak D.J. (2006). Analysis of *Pseudomonas aeruginosa* Conditional Psl Variants Reveals Roles for the Psl Polysaccharide in Adhesion and Maintaining Biofilm Structure Postattachment. J. Bacteriol..

[154] Franklin M.J., Nivens D.E., Weadge J.T., Howell P.L. (2011). Biosynthesis of the *Pseudomonas aeruginosa* Extracellular Polysaccharides, Alginate, Pel, and Psl. Front. Microbiol..

[155] Friedman L., Kolter R. (2004). Genes involved in matrix formation in *Pseudomonas aeruginosa* PA14 biofilms. Mol. Microbiol..

[156] Gilbert K.B., Kim T.H., Gupta R., Greenberg E.P., Schuster M. (2009). Global position analysis of the *Pseudomonas aeruginosa* quorum-sensing transcription factor LasR. Mol. Microbiol..

[157] Sakuragi Y., Kolter R. (2007). Quorum-Sensing Regulation of the Biofilm Matrix Genes (pel) of *Pseudomonas aeruginosa*. J. Bacteriol..

[158] Wright G.D. (2010). Antibiotic resistance in the environment: A link to the clinic?. Curr. Opin. Microbiol..

[159] Pang Z., Raudonis R., Glick B.R., Lin T.-J., Cheng Z. (2018). Antibiotic resistance in *Pseudomonas aeruginosa:* Mechanisms and alternative therapeutic strategies. Biotechnol. Adv..

[160] Galdino A.C.M., Viganor L., de Castro A.A., da Cunha E.F.F., Mello T.P., Mattos L.M., Pereira M.D., Hunt M.C., O’Shaughnessy M., Howe O. (2019). Disarming *Pseudomonas aeruginosa* Virulence by the Inhibitory Action of 1,10-Phenanthroline-5,6-Dione-Based Compounds: Elastase B (LasB) as a Chemotherapeutic Target. Front. Microbiol..

[161] Evans A., Kavanagh K.A.J.J.o.M.M. (2021). Evaluation of metal-based antimicrobial compounds for the treatment of bacterial pathogens. J. Med. Microbiol..

[162] Frei A. (2020). Metal complexes, an untapped source of antibiotic potential?. Antibiotics.

[163] Lin D.M., Koskella B., Lin H.C. (2017). Phage therapy: An alternative to antibiotics in the age of multi-drug resistance. World J. Gastrointest. Pharmacol. Ther..

[164] Cotter P.D., Ross R.P., Hill C. (2013). Bacteriocins—A viable alternative to antibiotics?. Nat. Rev. Microbiol..

[165] Silva A., Silva V., Igrejas G., Poeta P. (2020). Carbapenems and *Pseudomonas aeruginosa:* Mechanisms and epidemiology. Antibiotics and Antimicrobial Resistance Genes in the Environment.

[166] Blair J.M.A., Webber M.A., Baylay A.J., Ogbolu D.O., Piddock L.J.V. (2015). Molecular mechanisms of antibiotic resistance. Nat. Rev. Microbiol..

[167] Hancock R.E.W. (1998). Resistance Mechanisms in *Pseudomonas aeruginosa* and Other Nonfermentative Gram-Negative Bacteria. Clin. Infect. Dis..

[168] Delcour A.H. (2009). Proteomics. Outer membrane permeability and antibiotic resistance. Biochim. Biophys. Acta Proteins Proteom..

[169] Hancock R.E.W., Brinkman F.S.L. (2002). Function of Pseudomonas Porins in Uptake and Efflux. Annu. Rev. Microbiol..

[170] Tamber S., Hancock R.E. (2003). On the mechanism of solute uptake in Pseudomonas. Front Biosci..

[171] E El Zowalaty M., A Al Thani A., Webster T.J., Schweizer H.P., Nasrallah G.K., E Marei H., Ashour H.M. (2015). *Pseudomonas aeruginosa:* Arsenal of resistance mechanisms, decades of changing resistance profiles, and future antimicrobial therapies. Futur. Microbiol..

[172] Marquez B. (2005). Bacterial efflux systems and efflux pumps inhibitors. Biochimie.

[173] Venter H., Mowla R., Ohene-Agyei T., Ma S. (2015). RND-type drug efflux pumps from Gram-negative bacteria: Molecular mechanism and inhibition. Front. Microbiol..

[174] Lister P.D., Wolter D.J., Hanson N.D. (2009). Antibacterial-Resistant *Pseudomonas aeruginosa:* Clinical Impact and Complex Regulation of Chromosomally Encoded Resistance Mechanisms. Clin. Microbiol. Rev..

[175] Aendekerk S., Diggle S.P., Song Z., Høiby N., Cornelis P., Williams P., Cámara M. (2005). The MexGHI-OpmD multidrug efflux pump controls growth, antibiotic susceptibility and virulence in *Pseudomonas aeruginosa* via 4-quinolone-dependent cell-to-cell communication. Microbiology.

[176] Wright G.D. (2005). Bacterial resistance to antibiotics: Enzymatic degradation and modification. Adv. Drug Deliv. Rev..

[177] Bush K., Jacoby G.A. (2010). Updated Functional Classification of β-Lactamases. Antimicrob. Agents Chemother..

[178] Juan C., Torrens G., González-Nicolau M., Oliver A. (2017). Diversity and regulation of intrinsic β-lactamases from non-fermenting and other Gram-negative opportunistic pathogens. FEMS Microbiol. Rev..

[179] Rawat D., Nair D. (2010). Extended-spectrum β-lactamases in Gram Negative Bacteria. J. Glob. Infect. Dis..

[180] Kumar P., Kizhakkedathu J.N., Straus S.K. (2018). Antimicrobial Peptides: Diversity, Mechanism of Action and Strategies to Improve the Activity and Biocompatibility In Vivo. Biomolecules.

[181] Zhou J., Zhang L., He Y., Liu K., Zhang F., Zhang H., Lu Y., Yang C., Wang Z., Fareed M.S. (2021). An optimized analog of antimicrobial peptide Jelleine-1 shows enhanced antimicrobial activity against multidrug resistant *P. aeruginosa* and negligible toxicity in vitro and in vivo. Eur. J. Med. Chem..

[182] Grassi L., Batoni G., Ostyn L., Rigole P., Bossche S.V.D., Rinaldi A., Maisetta G., Esin S., Coenye T., Crabbe A. (2019). The Antimicrobial Peptide lin-SB056-1 and Its Dendrimeric Derivative Prevent *Pseudomonas aeruginosa* Biofilm Formation in Physiologically Relevant Models of Chronic Infections. Front. Microbiol..

[183] Parducho K.R., Beadell B., Ybarra T.K., Bush M., Escalera E., Trejos A.T., Chieng A., Mendez M., Anderson C., Park H. (2020). The Antimicrobial Peptide Human Beta-Defensin 2 Inhibits Biofilm Production of *Pseudomonas aeruginosa* without Compromising Metabolic Activity. Front. Immunol..

[184] Jones R.A., Shropshire H., Zhao C., Murphy A., Lidbury I., Wei T., Scanlan D.J., Chen Y. (2021). Phosphorus stress induces the synthesis of novel glycolipids in *Pseudomonas aeruginosa* that confer protection against a last-resort antibiotic. ISME J..

[185] Breidenstein E.B., de la Fuente-Núñez C., Hancock R.E. (2011). *Pseudomonas aeruginosa:* All roads lead to resistance. Trends Microbiol..

[186] Rodriguez-Valera F., Martin-Cuadrado A.-B., López-Pérez M. (2016). Flexible genomic islands as drivers of genome evolution. Curr. Opin. Microbiol..

[187] Botelho J., Grosso F., Peixe L. (2019). Antibiotic resistance in *Pseudomonas aeruginosa*—Mechanisms, epidemiology and evolution. Drug Resist. Updat..

[188] Umadevi S., Joseph N.M., Kumari K., Easow J.M., Kumar S., Stephen S., Srirangaraj S., Raj S. (2011). Detection of extended spectrum beta lactamases, ampc beta lactamases and metallobetalactamases in clinical isolates of ceftazidime resistant *Pseudomonas aeruginosa*. Braz. J. Microbiol..

[189] Lopatkin A.J., Meredith H.R., Srimani J.K., Pfeiffer C., Durrett R., You L. (2017). Persistence and reversal of plasmid-mediated antibiotic resistance. Nat. Commun..

[190] Bennett P.M. (2008). Plasmid encoded antibiotic resistance: Acquisition and transfer of antibiotic resistance genes in bacteria. Br. J. Pharmacol..

[191] Liu Y.-Y., Wang Y., Walsh T., Yi L.-X., Zhang R., Spencer J., Doi Y., Tian G., Dong B., Huang X. (2015). Emergence of plasmid-mediated colistin resistance mechanism MCR-1 in animals and human beings in China: A microbiological and molecular biological study. Lancet Infect. Dis..

[192] Munita J.M., Arias C.A. (2016). Mechanisms of antibiotic resistance. Microbiol. Spectr..

[193] Kao C.-Y., Chen S.-S., Hung K.-H., Wu H.-M., Hsueh P.-R., Yan J.-J., Wu J.-J. (2016). Overproduction of active efflux pump and variations of OprD dominate in imipenem-resistant *Pseudomonas aeruginosa* isolated from patients with bloodstream infections in Taiwan. BMC Microbiol..

[194] Köhler T., Epp S.F., Curty L.K., Pechère J.-C. (1999). Characterization of MexT, the Regulator of the MexE-MexF-OprN Multidrug Efflux System of *Pseudomonas aeruginosa*. J. Bacteriol..

[195] Berrazeg M., Jeannot K., Enguéné V.Y.N., Broutin I., Loeffert S., Fournier D., Plésiat P. (2015). Mutations in β-Lactamase AmpC Increase Resistance of *Pseudomonas aeruginosa* Isolates to Antipseudomonal Cephalosporins. Antimicrob. Agents Chemother..

[196] Fernández L., Breidenstein E.B., Hancock R.E. (2011). Creeping baselines and adaptive resistance to antibiotics. Drug Resist. Updat..

[197] Stewart P.S.J.I.j.o.m.m. (2002). Mechanisms of antibiotic resistance in bacterial biofilms. Int. J. Med Microbiol..

[198] Wilton M., Charron-Mazenod L., Moore R., Lewenza S. (2016). Extracellular DNA Acidifies Biofilms and Induces Aminoglycoside Resistance in *Pseudomonas aeruginosa*. Antimicrob. Agents Chemother..

[199] Goli H.R., Nahaei M.R., Rezaee M.A., Hasani A., Kafil H.S., Aghazadeh M. (2016). Emergence of colistin resistant *Pseudomonas aeruginosa* at Tabriz hospitals, Iran. Iran. J. Microbiol..

[200] Azimi L., Lari A.R. (2019). Colistin-resistant *Pseudomonas aeruginosa* clinical strains with defective biofilm formation. GMS Hyg. Infect. Control..

[201] Haenni M., Bour M., Châtre P., Madec J.-Y., Plésiat P., Jeannot K. (2017). Resistance of Animal Strains of *Pseudomonas aeruginosa* to Carbapenems. Front. Microbiol..

[202] Castanon J.I.R. (2007). History of the Use of Antibiotic as Growth Promoters in European Poultry Feeds. Poult. Sci..

[203] Wanty C., Anandan A., Piek S., Walshe J., Ganguly J., Carlson R.W., Stubbs K.A., Kahler C.M., Vrielink A. (2013). The Structure of the Neisserial Lipooligosaccharide Phosphoethanolamine Transferase A (LptA) Required for Resistance to Polymyxin. J. Mol. Biol..

[204] Andrade F.F., Silva D., Rodrigues A., Pina-Vaz C. (2020). Colistin Update on Its Mechanism of Action and Resistance, Present and Future Challenges. Microorganisms.

[205] Dößelmann B., Willmann M., Steglich M., Bunk B., Nübel U., Peter S., Neher R.A. (2017). Rapid and Consistent Evolution of Colistin Resistance in Extensively Drug-Resistant *Pseudomonas aeruginosa* during Morbidostat Culture. Antimicrob. Agents Chemother..

[206] Arcilla M.S., van Hattem J.M., Matamoros S., Melles D.C., Penders J., de Jong M.D., Schultsz C. (2016). Dissemination of the mcr-1 colistin resistance gene. Lancet.

[207] Lima T., Domingues S., Da Silva G.J. (2019). Plasmid-Mediated Colistin Resistance in Salmonella enterica: A Review. Microorganisms.

[208] Pesingi P.V., Singh B.R., Pesingi P.K., Bhardwaj M., Singh S.V., Kumawat M., Sinha D.K., Gandham R.K. (2019). MexAB-OprM Efflux Pump of *Pseudomonas aeruginosa* Offers Resistance to Carvacrol: A Herbal Antimicrobial Agent. Front. Microbiol..

[209] Moskowitz S.M., Ernst R., Miller S.I. (2004). PmrAB, a Two-Component Regulatory System of *Pseudomonas aeruginosa* That Modulates Resistance to Cationic Antimicrobial Peptides and Addition of Aminoarabinose to Lipid A. J. Bacteriol..

[210] Macfarlane E.L.A., Kwasnicka A., Hancock R.E.W. (2000). Role of *Pseudomonas aeruginosa* PhoP-PhoQ in resistance to antimicrobial cationic peptides and aminoglycosides. Microbiology.

[211] Ezadi F., Ardebili A., Mirnejad R. (2019). Antimicrobial Susceptibility Testing for Polymyxins: Challenges, Issues, and Recommendations. J. Clin. Microbiol..

[212] McPhee J., Bains M., Winsor G., Lewenza S., Kwasnicka A., Brazas M., Brinkman F.S.L., Hancock R.E.W. (2006). Contribution of the PhoP-PhoQ and PmrA-PmrB Two-Component Regulatory Systems to Mg^2+^ -Induced Gene Regulation in *Pseudomonas aeruginosa*. J. Bacteriol..

[213] Fernández L., Gooderham W.J., Bains M., McPhee J., Wiegand I., Hancock R.E.W. (2010). Adaptive Resistance to the “Last Hope” Antibiotics Polymyxin B and Colistin in *Pseudomonas aeruginosa* Is Mediated by the Novel Two-Component Regulatory System ParR-ParS. Antimicrob. Agents Chemother..

